# Trends in Mammography Use Among Women Aged 40 to 74 Years in the US, 2002-2022

**DOI:** 10.1001/jamanetworkopen.2026.3529

**Published:** 2026-03-26

**Authors:** Syed Mahfuz Al Hasan, Debbie L. Bennett, Adetunji T. Toriola

**Affiliations:** 1 Division of Public Health Sciences, Department of Surgery, Washington University School of Medicine, St Louis, Missouri; 2 Mallinckrodt Institute of Radiology, Washington University School of Medicine, St Louis, Missouri; 3 Siteman Cancer Center, Washington University School of Medicine, St Louis, Missouri

## Abstract

**Importance:**

Understanding how prior policies, systemic factors, and public health events have shaped mammography use among US women is essential for interpreting the 2024 US Preventive Services Task Force (USPSTF) recommendation update and identifying gaps to inform equitable public health strategies.

**Objective:**

To analyze mammography use trends in US women aged 40 to 49 and 50 to 74 years from 2002 to 2022 and assess variation by sociodemographic, health care access, behavioral, and geographic factors, including associations with the 2009 USPSTF recommendation and the COVID-19 pandemic.

**Design, Setting, and Participants:**

This cross-sectional study used data from the Behavioral Risk Factor Surveillance System biennial cancer screening module (2002-2022). Joinpoint regression with a jump model was used to account for methodological changes in the Behavioral Risk Factor Surveillance System beginning in 2011. Data were analyzed from March to September 2025.

**Main Outcomes and Measures:**

The primary outcome was the average biennial percentage change in mammography use from 2002 to 2022, calculated as a weighted average of jump model–estimated biennial changes. Subgroup analyses were conducted by sociodemographic, health care access, behavioral, and geographic factors.

**Results:**

Among 2 619 292 US women, mammography use showed a downward trend, with nonsignificant biennial changes of −0.54% (95% CI, −1.25% to 0.16%) for ages 40 to 49 years and −0.16% (95% CI, −0.49% to 0.16%) for ages 50 to 74 years from 2002 to 2022. Declines were significant among young women without health insurance (–1.54%; 95% CI, –2.89% to –0.17%), current smokers (–1.36%; 95% CI, –2.43% to –0.27%), unmarried women (–1.10%; 95% CI, –1.61% to –0.59%), and non-Hispanic White women (–0.58%; 95% CI, –1.09% to –0.07%). Following the 2009 USPSTF recommendation, mammography use decreased significantly among younger non-Hispanic White (−0.88%; 95% CI, −1.59% to −0.16%), Asian (−2.45%; 95% CI, −4.20% to −0.25%), and uninsured (−2.39%; 95% CI, −4.57% to −0.16%) women, with no significant reduction among non-Hispanic Black women (−0.59%; 95% CI, −1.20% to 0.03%). Across both age groups, mammography use was consistently lower in Western than in Eastern states.

**Conclusions and Relevance:**

In this cross-sectional study, mammography use did not significantly decline in the overall population; however, significant reductions were observed among non-Hispanic White, Asian, and uninsured women aged 40 to 49 years from 2010 to 2022. These findings highlight the need for clear, risk-based screening communication and targeted strategies to promote guideline-concordant decision-making, particularly among younger women.

## Key Points

**Question:**

How has mammography use among US women changed over time?

**Findings:**

In this cross-sectional study of more than 2 million US women, mammography use did not significantly decline in the overall population from 2002 to 2022. However, biennial declines were significant among non-Hispanic White women aged 40 to 49 years, uninsured women, and current smokers.

**Meaning:**

Declining mammography use among subgroups of younger US women underscores the need for clear, risk-based screening communication and targeted strategies to support guideline-concordant decision-making.

## Teaser

This cross-sectional study analyzes mammography use trends in US women aged 40 to 74 years from 2002 to 2022 and assesses variation by sociodemographic, health care access, behavioral, and geographic factors.

## Introduction

Breast cancer is the most commonly diagnosed cancer and the second leading cause of cancer-related death among US women, accounting for an estimated 32% of all new cancer cases and 42 170 deaths in 2025.^1^ Given its high incidence and mortality burden, effective strategies for early detection are critical to reducing breast cancer–related deaths.^2,3^ Regular mammography screening remains the cornerstone of early detection and timely treatment.^4,5^

Over the past 2 decades, breast cancer screening guidelines in the US have undergone several evidence-based revisions. In 2009, the US Preventive Services Task Force (USPSTF), on the basis of systematic evaluation of benefits and harms, recommended against routine screening for women aged 40 to 49 years and emphasized biennial screening for women aged 50 to 74 years.^6^ In 2024, incorporating updated evidence, the USPSTF updated its guidance to recommend biennial screening for women aged 40 to 74 years.^7^ The American Cancer Society’s 2015 guidelines recommended initiating screening at age 45 years, with the option to begin at age 40 years according to individual preference and informed decision-making.^8^ In subsequent years, the COVID-19 pandemic disrupted access to preventive services, including breast cancer screening, potentially exacerbating existing barriers to care.^9,10,11,12^

Few studies have comprehensively evaluated national trends in mammography use. To our knowledge, only 1 study to date has evaluated the association between the 2009 USPSTF recommendation and changes in mammography screening using Behavioral Risk Factor Surveillance System (BRFSS) data from 2000 to 2018, excluding 2020 and 2022 because of COVID-19 pandemic–related data issues.^13^ Consequently, a critical gap remains in understanding mammography use trends amid evolving screening guidelines and health system disruptions, as major BRFSS methodological changes implemented in 2011, including cellular telephone sampling and raking-based weighting, may have substantially influenced prevalence estimates and were not addressed in prior trend analyses.^14^

Using 2 decades of nationally representative BRFSS data, accounting for major methodological changes, and disaggregating trends by age, race and ethnicity, socioeconomic characteristics, and geography, this study provides a comprehensive assessment of mammography use among US women aged 40 to 49 and 50 to 74 years from 2002 to 2022. We further evaluated changes in mammography use following the 2009 USPSTF breast cancer screening recommendation.

## Methods

### Data Source and Compilation

We used publicly available and repeated cross-sectional population health survey data from the Centers for Disease Control and Prevention’s BRFSS,^15^ which is the world’s largest ongoing telephone-based health survey of noninstitutionalized US adults. BRFSS collects information on health behaviors, chronic conditions, health care access, and use of preventive services across all 50 states, the District of Columbia, and participating territories.^16^ It serves as a key tool for monitoring population health trends and informing policy.^17^

The BRFSS breast cancer screening module is implemented in even-numbered years; therefore, we compiled data for all biennial cycles from 2002 to 2022 to construct a harmonized dataset for trend analysis. We systematically reviewed each dataset to identify variable availability, naming conventions, and coding structures, standardizing conceptually equivalent variables—including income, employment, marital status, and race and ethnicity—across survey years. This harmonization ensured consistent variable definitions and coding, enabling valid comparisons of mammography use trends over time. As all data were publicly available and deidentified, and the study did not involve human participants, institutional review board review or exemption was not required under the Common Rule (45 CFR §46). This study adhered to the Strengthening the Reporting of Observational Studies in Epidemiology (STROBE) reporting guidelines for cross-sectional studies.^18^

### Mammography Use Data

This study included female respondents aged 40 to 74 years who reported a mammogram within the past 2 years.^19^ Eligibility was based on participants’ responses to whether they had ever had a mammogram (BRFSS variable HADMAM) and the time since their most recent examination (BRFSS variable HOWLONG).^19^ As BRFSS relies on self-reported data and does not distinguish between screening and diagnostic mammograms, we refer to this outcome as *mammography use* throughout the article. We excluded individuals outside the target age range (ie, <40 or >74 years), male respondents, and those who responded do not know, not sure, or refused to answer the mammography-related questions.

### Subgroup Analysis

Subgroup trend analyses were conducted by demographic, socioeconomic, health care access, and health-related behavioral characteristics. Race and ethnicity were self-reported during the BRFSS interview. All reported categories were considered, but small sample sizes required combining some groups. Subgroup analysis focused on American Indian or Alaska Native, Asian, Hispanic or Latino, non-Hispanic Black, non-Hispanic White, and other (including Native Hawaiian or Other Pacific Islander, multiracial, and unspecified races and ethnicities labeled as *Others* in BRFSS). Socioeconomic measures included education (less than high school, high school graduate, some college, and college graduate), marital status (married, separated, and not married), employment (employed, out of work, homemaker, and other), and annual household income (<$25 000, $25 000-$49 999, and ≥$50 000). Health care access variables were insurance status (insured vs uninsured) and having a regular health care practitioner (yes vs no). Behavioral characteristics were self-reported and derived from BRFSS-computed variables, including smoking status (never, current, and former), physical activity (active vs inactive), and self-rated health (good vs poor). All variables were harmonized across survey cycles to ensure consistency and comparability over time. Detailed BRFSS question and response options are provided in eTable 1 in Supplement 1.

### Statistical Analysis

Data were analyzed from March to September 2025. We applied the BRFSS survey weights to account for the complex sampling design, including stratification, clustering, and unequal selection probabilities.^20^ Weighted estimates ensured representativeness of the noninstitutionalized US adult population. We calculated weighted prevalence of mammography use and 95% CIs for women aged 40 to 49 and 50 to 74 years, stratified by demographic, socioeconomic, health care access, and behavioral characteristics, as well as by state. Survey weights also adjust for differential selection probabilities and potential nonresponse bias in the BRFSS design. For trend analyses, we compiled a harmonized dataset containing weighted mammography prevalence for each age group, subgroup, and state across all biennial survey years from 2002 to 2022. Statistical significance was defined as a 95% CI that did not include the null value of zero.

#### Trend Analysis

Joinpoint regression analysis^21^ was used to examine trends in mammography use among women aged 40 to 49 years and 50 to 74 years, stratified by demographic, socioeconomic, health care access, behavioral characteristics, and state. Model selection was guided by a data-driven approach using the bayesian information criterion, with the optimal model defined as the one minimizing the weighted bayesian information criterion value.^22^ The model estimated average biennial percentage change (ABPC) and 95% CI, calculated as a geometric weighted average of segment-specific biennial percentage changes, providing insight into long-term trends from 2002 to 2022. Beginning in 2011, BRFSS expanded the sampling frame to include cellular telephone–only households and replaced traditional poststratification weights with iterative proportional fitting (raking).^14^ Although these changes improved population coverage and reduced noncoverage bias, they also introduced a potential discontinuity in prevalence estimates unrelated to true changes in mammography use.^14^ To address this, we incorporated a jump model in the joinpoint analysis, treating 2010 as the final prechange year and estimating a level shift in 2012.^23^ This approach allows subsequent slope estimates to be interpreted as underlying temporal trends, unconfounded by the 2011 methodological change. The joinpoint regression analysis was performed using the Windows-based Joinpoint Regression Program version 5.4.0.0 (National Institutes of Health).

#### Trend Comparison Following the 2009 USPSTF Recommendations

To assess changes in mammography use following the 2009 USPSTF recommendation, we conducted a comparative trend analysis, dividing the study period into prerecommendation (2002-2008) and postrecommendation (2010-2022) intervals. For each interval, we estimated the ABPC, calculated as a weighted average of segment-specific biennial changes from the jump model. This measure accounts for the duration of each segment and provides a summary indicator of the overall rate of change in mammography use during each period.

#### Mammography Use and the COVID-19 Pandemic

To examine changes in mammography use prevalence potentially associated with the COVID-19 pandemic, we compared the ABPC between 2012 and 2018 (prepandemic) and 2012 and 2022 (including pandemic years). The time frame was selected to include only post-2009 USPSTF observations while avoiding sudden shifts in mammography prevalence due to the BRFSS survey methodology change in 2011. For this analysis, ABPC estimates were derived using the standard joinpoint model. Analyses were conducted separately for women aged 40 to 49 and 50 to 74 years and were stratified by demographic, socioeconomic, health care access, and behavioral characteristics and by state.

## Results

### Trends in Mammography Use

**Figure 1.**
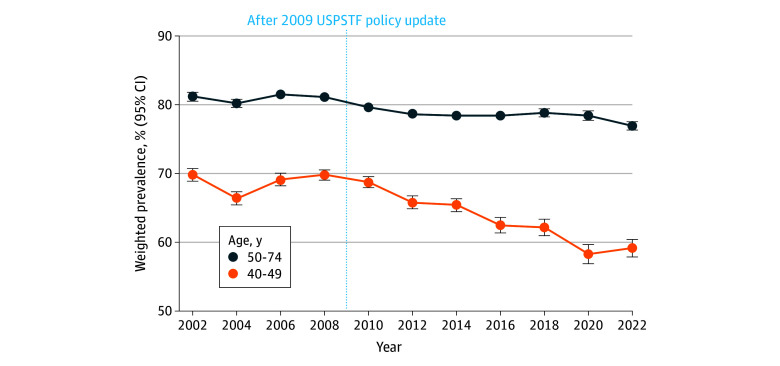
Line Graph of Trends in Mammography Use Prevalence Among Women Aged 40 to 49 and 50 to 74 Years in the US, 2002 to 2022 Error bars indicate the 95% CI of weighted prevalence. USPSTF indicates US Preventive Services Task Force.

Our analysis included 2 619 292 surveyed women with a 3.7% missing response on mammography use (eTable 2 in Supplement 1). Mammography use was consistently higher among women aged 50 to 74 years than among those aged 40 to 49 years from 2002 to 2022 (Figure 1). However, prevalence declined in both age groups, from 81.3% (95% CI, 80.6% to 81.9%) in 2002 to 77.0% (95% CI, 76.4% to 77.6%) in 2022 among women aged 50 to 74 years, and from 69.9% (95% CI, 68.9% to 70.8%) in 2002 to 59.2% (95% CI, 57.9% to 60.4%) in 2022 among women aged 40 to 49 years. Biennial changes from 2002 to 2022 were not statistically significant for women aged 50 to 74 years (−0.16%; 95% CI, −0.49% to 0.16%) and women aged 40 to 49 years (−0.54%; 95% CI, −1.25% to 0.16%) (Table 1). Across the study years, mammography use increased with higher levels of educational attainment among women aged 40 to 49 and 50 to 74 years, with a constantly higher prevalence among college graduates compared with women with less than a high school education (eTables 3 and 4 in Supplement 1). A similar lower mammography use prevalence was observed among women with the lowest income, uninsured women, and those without a health care practitioner.

**Table 1.** Prevalence and Biennial Changes in Mammography Use Among Women in the US, 2002-2022

Characteristics	Age 40-49 y			Age 50-74 y		
	Prevalence, % (95% CI)^a^		2002-2022, ABPC (95% CI)^b^	Prevalence, % (95% CI)^a^		2002-2022, ABPC (95% CI)^b^
	BRFSS 2002	BRFSS 2022		BRFSS 2002	BRFSS 2022	
Overall	69.9 (68.9 to 70.8)	59.2 (57.9 to 60.4)	−0.54 (−1.25 to 0.16)	81.3 (80.6 to 81.9)	77.0 (76.4 to 77.6)	−0.16 (−0.49 to 0.16)
Race and ethnicity						
American Indian or Alaska Native	63.2 (54.1 to 71.4)	55.2 (45.8 to 64.2)	−0.06 (−0.95 to 0.84)	76.8 (68.4 to 83.5)	62.1 (55.8 to 68.1)	−0.78 (−2.49 to 0.95)
Asian	60.4 (50.0 to 69.9)	55.4 (48.4 to 62.3)	−1.44 (−4.70 to 1.93)	71.4 (58.4 to 81.6)	76.0 (70.5 to 80.8)	0.02 (−2.09 to 2.19)
Hispanic or Latino	63.1 (58.9 to 67.2)	55.1 (51.6 to 58.5)	−0.07 (−1.52 to 1.41)	78.6 (74.7 to 82.1)	74.9 (72.5 to 77.1)	0.04 (−0.87 to 0.96)
Non-Hispanic Black	72.3 (69.3 to 75.1)	64.9 (61.8 to 67.9)	−0.27 (−0.88 to 0.35)	83.1 (80.7 to 85.2)	83.3 (81.8 to 84.6)	0.04 (−0.18 to 0.26)
Non-Hispanic White	71.3 (70.3 to 72.2)	60.4 (59.0 to 61.7)	−0.58 (−1.09 to −0.07)	81.7 (81.1 to 82.3)	77.0 (76.4 to 77.6)	−0.19 (−0.44 to 0.05)
Other race or multiracial^c^	66.2 (59.6 to 72.2)	54.7 (48.2 to 61.0)	−1.32 (−2.65 to 0.03)	79.1 (73.3 to 83.9)	66.1 (60.6 to 71.3)	−0.44 (−1.37 to 0.50)
Education						
Below high school	56.3 (51.7 to 60.9)	45.1 (40.0 to 50.2)	−0.87 (−3.62 to 1.95)	71.4 (68.9 to 73.8)	65.6 (62.7 to 68.4)	−0.05 (−1.15 to 1.06)
High school graduate	66.7 (65.0 to 68.3)	56.1 (53.2 to 58.9)	−1.15 (−1.91 to −0.39)	80.8 (79.7 to 81.7)	74.3 (73.1 to 75.4)	−0.40 (−0.65 to −0.15)
Some college	71.7 (70.0 to 73.4)	56.0 (53.7 to 58.2)	−1.02 (−1.58 to −0.47)	82.2 (81.1 to 83.3)	77.2 (76.2 to 78.2)	−0.28 (−0.46 to −0.10)
College graduate	75.5 (74.0 to 77.0)	67.1 (65.6 to 68.6)	−0.64 (−1.11 to −0.16)	86.4 (85.1 to 87.6)	81.8 (80.9 to 82.6)	−0.22 (−0.37 to −0.07)
Employment						
Employed for wages^d^	71.8 (70.7 to 72.9)	60.6 (59.2 to 62.1)	−0.55 (−1.05 to −0.05)	82.1 (81.2 to 83.0)	77.4 (76.4 to 78.3)	−0.20 (−0.33 to −0.07)
Out of work^e^	60.7 (56.2 to 65.1)	56.6 (51.6 to 61.5)	−0.06 (−1.59 to 1.51)	74.6 (70.9 to 78.0)	66.9 (63.4 to 70.3)	−0.36 (−0.56 to −0.15)
Homemaker	64.5 (61.6 to 67.2)	55.2 (51.2 to 59.1)	−0.75 (−1.38 to −0.11)	79.5 (77.5 to 81.2)	71.3 (68.6 to 73.9)	−0.41 (−0.71 to −0.11)
Others^f^	67.3 (63.0 to 71.3)	53.5 (49.5 to 57.4)	−0.85 (−2.25 to 0.56)	81.5 (80.4 to 82.5)	78.0 (77.1 to 78.9)	−0.08 (−0.47 to 0.32)
Annual household income, $						
<25 000	58.0 (55.5 to 60.4)	49.6 (46.5 to 52.7)	−0.70 (−2.03 to 0.65)	73.5 (72.1 to 74.8)	67.0 (65.1 to 68.8)	−0.18 (−0.71 to 0.35)
25 000 to 49 999	68.2 (66.4 to 70.0)	53.4 (50.4 to 56.3)	−1.26 (−1.94 to −0.57)	83.2 (82.1 to 84.3)	74.0 (72.6 to 75.3)	−0.52 (−0.78 to −0.26)
≥50 000	76.9 (75.5 to 78.2)	63.7 (62.1 to 65.3)	−0.77 (−1.12 to −0.43)	87.5 (86.3 to 88.5)	81.3 (80.5 to 82.1)	−0.31 (−0.44 to −0.19)
Marital status						
Married	72.2 (71.0 to 73.3)	62.1 (60.5 to 63.7)	−0.55 (−1.07 to −0.03)	83.8 (82.9 to 84.6)	80.0 (79.3 to 80.8)	−0.17 (−0.44 to 0.11)
Separated^g^	65.6 (63.6 to 67.6)	56.1 (53.6 to 58.6)	−0.46 (−1.04 to 0.13)	77.3 (76.2 to 78.4)	72.0 (70.9 to 73.1)	−0.21 (−0.60 to 0.18)
Not married^h^	64.1 (61.1 to 67.0)	52.4 (49.8 to 55.0)	−1.10 (−1.61 to −0.59)	76.2 (73.3 to 78.9)	73.1 (71.1 to 75.0)	−0.15 (−0.58 to 0.28)
Insurance						
Have insurance	73.7 (72.7 to 74.6)	61.9 (60.6 to 63.1)	−0.84 (−1.27 to −0.40)	83.7 (83.0 to 84.3)	78.3 (77.7 to 78.9)	−0.27 (−0.47 to −0.08)
No insurance	46.7 (43.8 to 49.6)	33.2 (28.8 to 37.8)	−1.54 (−2.89 to −0.17)	58.1 (55.2 to 60.9)	38.2 (34.5 to 42.1)	−0.81 (−2.42 to 0.84)
Primary HCP						
Have an HCP	73.9 (72.9 to 74.8)	63.3 (62.0 to 64.6)	−0.64 (−1.08 to −0.20)	84.3 (83.7 to 84.9)	79.2 (78.6 to 79.8)	−0.21 (−0.53 to 0.11)
No HCP	44.9 (41.9 to 48.0)	32.5 (29.4 to 35.8)	−1.49 (−3.12 to 0.17)	51.4 (47.9 to 54.8)	43.2 (40.0 to 46.4)	−0.69 (−1.84 to 0.46)
General health						
Good health	70.7 (69.7 to 71.7)	60.9 (59.6 to 62.3)	−0.54 (−1.03 to −0.05)	82.6 (81.9 to 83.3)	78.8 (78.1 to 79.5)	−0.15 (−0.47 to 0.18)
Poor health	65.1 (62.3 to 67.8)	51.0 (48.0 to 54.1)	−0.91 (−2.54 to 0.75)	76.8 (75.4 to 78.2)	70.0 (68.6 to 71.4)	−0.32 (−0.69 to 0.06)
Physical activity						
Yes	71.8 (70.7 to 72.8)	61.1 (59.7 to 62.5)	−0.66 (−1.14 to −0.17)	83.7 (82.9 to 84.4)	79.1 (78.4 to 79.7)	−0.14 (−0.48 to 0.21)
No	64.2 (62.1 to 66.3)	53.1 (50.5 to 55.7)	−0.68 (−1.62 to 0.27)	75.6 (74.3 to 76.8)	71.1 (69.8 to 72.4)	−0.25 (−0.64 to 0.13)
Smoking status						
Current smoker	60.3 (58.4 to 62.2)	46.5 (43.7 to 49.4)	−1.36 (−2.43 to −0.27)	69.2 (67.2 to 71.1)	63.4 (61.7 to 65.1)	−0.52 (−0.76 to −0.27)
Former smoker	73.7 (71.6 to 75.7)	60.4 (57.8 to 63.0)	−0.89 (−1.43 to −0.36)	84.4 (83.3 to 85.4)	76.4 (75.2 to 77.6)	−0.32 (−0.81 to 0.16)
Never smoked	72.7 (71.4 to 74.0)	61.7 (60.1 to 63.3)	−0.51 (−1.20 to 0.19)	83.4 (82.5 to 84.2)	80.1 (79.3 to 80.9)	−0.11 (−0.37 to 0.15)
Alcohol intake						
Yes	72.9 (71.7 to 74.2)	61.8 (60.1 to 63.4)	−0.52 (−1.00 to −0.05)	85.0 (84.0 to 85.8)	80.0 (79.1 to 80.8)	−0.15 (−0.58 to 0.29)
No	66.6 (65.1 to 68.1)	56.1 (54.1 to 58.0)	−0.78 (−1.29 to −0.26)	78.7 (77.8 to 79.6)	74.4 (73.5 to 75.3)	−0.17 (−0.52 to 0.18)

Abbreviations: ABPC, average biennial percentage change; BRFSS, Behavioral Risk Factor Surveillance System; HCP, health care practitioner.

^a^ Weighted screening prevalence adjusted for the complex survey design.

^b^ Geometric weighted average of biennial percentage changes of segments of mammography use prevalence trends in the US (adjusted for changes in the BRFSS survey method in 2011).

^c^ Native Hawaiian or Other Pacific Islander and races and ethnicities not specified in the BRFSS dataset.

^d^ Employed for wages includes the employed for wages and the self-employed.

^e^ Out of work group included women who were out for work (<1 year and >1 year).

^f^ Other employment groups include students, the retired, and women unable to work.

^g^ Separated includes divorced, widowed, and separated women.

^h^ Not married includes never married and a member of an unmarried couple.

Declines in mammography use varied by race, education, employment, income, smoking status, and health care access, particularly among women aged 40 to 49 years. In this age group, non-Hispanic White women experienced a significant biennial decline (−0.58%; 95% CI, −1.09% to −0.07%), with prevalence decreasing from 71.3% (95% CI, 70.3% to 72.2%) in 2002 to 60.4% (95% CI, 59.0% to 61.7%) in 2022. In contrast, the decline in non-Hispanic Black women was not statistically significant (−0.27%; 95% CI, −0.88% to 0.35%), with prevalence decreasing from 72.3% (95% CI, 69.3% to 75.1%) in 2002 to 64.9% (95% CI, 61.8% to 67.9%) in 2022 (Table 1). Among women aged 50 to 74 years, mammography use did not decline significantly by race. However, across both age groups, declines were significant among women with at least a high school education and incomes greater than or equal to $25 000. Mammography use reduced significantly among women with a health care practitioner (−0.64%; 95% CI, −1.08% to −0.20%) and was more pronounced among uninsured women aged 40 to 49 years (−1.54%; 95% CI, −2.89% to −0.17%) and unmarried women (–1.10%; 95% CI, –1.61% to –0.59%). In contrast, declines in the 50- to 74-year-old age group were significant primarily among insured women (−0.27%; 95% CI, −0.47% to −0.08%). Current smokers experienced significant declines in mammography use among women aged 40 to 49 years (−1.36%; 95% CI, −2.43% to −0.27%) and 50 to 74 years (−0.52%; 95% CI, −0.76% to −0.27%).

### Statewide Trends in Mammography Use

Mammography use showed notable geographic variations, with lower prevalence in the Western US—particularly in the Rocky Mountain and Southwest regions—compared with the Eastern US (eFigures 1-3 in Supplement 1). Most states experienced a downward trend in mammography use from 2002 to 2022, varying by age group (eFigure 4 and eTables 5 and 6 in Supplement 1). Among women aged 40 to 49 years, declines were observed in 46 states and Washington, DC, but were statistically significant in only 7 states and Washington, DC, with the largest reductions in Vermont (−1.75%; 95% CI, −2.32% to −1.17%) and New Mexico (−1.59%; 95% CI, −2.37% to −0.81%). In contrast, South Dakota (1.37%; 95% CI, 0.12% to 2.63%) and Mississippi (0.93%; 95% CI, 0.32% to 1.54%) showed significant increases (Table 2). Among women aged 50 to 74 years, overall declines occurred in 43 states and Washington, DC, with the largest significant reductions in Alaska (−0.95%; 95% CI, −1.59% to −0.29%), followed by Colorado (−0.53%; 95% CI, −0.79% to −0.26%), Kansas (−0.51%; 95% CI, −0.82% to −0.19%), Vermont (−0.47%; 95% CI, −0.85% to −0.08%), and Washington, DC (−0.45%; 95% CI, −0.85% to −0.05%) (Table 2).

**Table 2.** Prevalence and Biennial Changes in Mammography Use by State, 2002-2022

State	Age 40-49 y			Age 50-74 y		
	Prevalence, % (95% CI)^a^		2002-2022, ABPC (95% CI)^b^	Prevalence, % (95% CI)^a^		2002-2022, ABPC (95% CI)^b^
	BRFSS 2002	BRFSS 2022		BRFSS 2002	BRFSS 2022	
Alabama	78.1 (73.2 to 82.3)	61.6 (53.0 to 69.4)	−0.68 (−1.70 to 0.34)	81.2 (77.9 to 84.2)	76.4 (73.0 to 79.4)	−0.36 (−0.80 to 0.09)
Alaska	59.9 (52.2 to 67.1)	53.9 (47.2 to 60.4)	−0.60 (−2.15 to 0.96)	82.2 (75.6 to 87.3)	68.7 (64.9 to 72.4)	−0.95 (−1.59 to −0.29)
Arizona	70.6 (63.4 to 76.9)	54.7 (48.4 to 61.0)	−0.29 (−2.41 to 1.87)	82.6 (78.8 to 85.8)	74.9 (72.1 to 77.4)	−0.30 (−0.63 to 0.02)
Arkansas	67.9 (63.0 to 72.4)	59.7 (53.1 to 66.0)	−0.41 (−1.49 to 0.69)	70.4 (66.9 to 73.6)	75.0 (72.0 to 77.7)	0.95 (−0.45 to 2.36)
California	66.3 (60.8 to 71.4)	52.4 (46.7 to 58.1)	−1.14 (−2.40 to 0.14)	81.4 (77.1 to 85.0)	76.5 (73.4 to 79.4)	−0.33 (−0.71 to 0.05)
Colorado	67.0 (62.1 to 71.5)	55.2 (50.6 to 59.7)	−0.76 (−1.72 to 0.22)	81.1 (78.0 to 83.9)	71.1 (68.2 to 74.0)	−0.53 (−0.79 to −0.26)
Connecticut	78.2 (74.1 to 81.8)	70.9 (65.6 to 75.7)	−0.40 (−0.50 to −0.30)	86.5 (83.6 to 88.9)	81.7 (79.0 to 84.0)	−0.09 (−0.51 to 0.33)
Delaware	78.4 (72.4 to 83.3)	61.4 (52.4 to 69.6)	−0.98 (−1.52 to −0.43)	88.8 (86.1 to 91.0)	79.7 (76.4 to 82.6)	−0.58 (−1.35 to 0.20)
District of Columbia	76.1 (68.2 to 82.6)	57.7 (49.9 to 65.1)	−1.31 (−1.90 to −0.71)	86.1 (81.7 to 89.6)	77.4 (72.7 to 81.6)	−0.45 (−0.85 to −0.05)
Florida	72.7 (68.8 to 76.3)	60.0 (52.5 to 67.1)	−0.43 (−3.19 to 2.41)	81.1 (78.3 to 83.5)	78.0 (74.9 to 80.8)	−0.01 (−1.08 to 1.06)
Georgia	69.1 (64.5 to 73.3)	60.6 (54.5 to 66.3)	−0.44 (−1.36 to 0.49)	80.6 (77.3 to 83.4)	76.3 (73.5 to 78.9)	−0.17 (−0.48 to 0.14)
Hawaii	63.7 (59.3 to 67.9)	65.4 (59.9 to 70.4)	−0.14 (−0.82 to 0.56)	76.1 (72.6 to 79.3)	78.5 (75.6 to 81.2)	0.34 (−0.32 to 1.01)
Idaho	59.3 (54.6 to 63.8)	48.7 (43.5 to 54.0)	−0.58 (−2.07 to 0.93)	73.0 (69.8 to 76.0)	68.5 (65.5 to 71.4)	−0.09 (−0.88 to 0.71)
Illinois	69.5 (62.7 to 75.5)	55.5 (48.8 to 62.0)	−0.96 (−2.56 to 0.67)	77.3 (72.7 to 81.4)	72.8 (68.4 to 76.8)	−0.36 (−1.07 to 0.36)
Indiana	67.1 (63.1 to 70.8)	58.1 (53.8 to 62.2)	−0.42 (−1.50 to 0.67)	79.6 (76.9 to 82.0)	78.1 (76.1 to 80.1)	0.37 (−0.87 to 1.62)
Iowa	70.2 (64.5 to 75.4)	58.3 (53.2 to 63.3)	−0.63 (−1.36 to 0.11)	83.1 (80.0 to 85.8)	79.7 (77.2 to 81.9)	−0.12 (−0.34 to 0.09)
Kansas	66.7 (62.4 to 70.7)	57.1 (52.3 to 61.8)	−0.77 (−1.14 to −0.39)	82.6 (79.9 to 85.1)	74.5 (72.1 to 76.7)	−0.51 (−0.82 to −0.19)
Kentucky	72.3 (67.5 to 76.7)	57.9 (49.8 to 65.5)	−1.01 (−2.54 to 0.54)	80.2 (77.7 to 82.6)	72.9 (68.6 to 76.8)	−0.46 (−1.65 to 0.74)
Louisiana	69.6 (64.8 to 74.1)	67.8 (61.4 to 73.5)	0.15 (−0.69 to 0.99)	80.9 (78.2 to 83.3)	82.5 (79.9 to 84.9)	0.31 (−0.01 to 0.64)
Maine	76.7 (71.3 to 81.3)	59.6 (54.4 to 64.5)	−1.25 (−1.70 to −0.80)	87.0 (83.6 to 89.7)	81.6 (79.6 to 83.5)	−0.23 (−0.58 to 0.12)
Maryland	79.2 (74.8 to 83.0)	66.4 (62.1 to 70.4)	−1.01 (−2.29 to 0.30)	85.9 (82.7 to 88.7)	83.2 (81.4 to 84.8)	−0.17 (−0.73 to 0.38)
Massachusetts	78.3 (74.9 to 81.3)	64.3 (59.8 to 68.5)	−1.28 (−1.8 to −0.77)	88.6 (86.4 to 90.5)	84.9 (82.8 to 86.9)	−0.24 (−0.44 to −0.04)
Michigan	72.1 (67.8 to 75.9)	66.2 (61.2 to 70.9)	−0.34 (−1.29 to 0.61)	83.3 (79.9 to 86.1)	77.7 (75.5 to 79.7)	−0.29 (−0.69 to 0.12)
Minnesota	74.8 (70.5 to 78.7)	62.0 (58.4 to 65.5)	−0.80 (−1.65 to 0.06)	86.5 (83.9 to 88.8)	79.6 (77.7 to 81.3)	−0.41 (−0.92 to 0.10)
Mississippi	60.6 (55.7 to 65.3)	65.4 (58.6 to 71.7)	0.93 (0.32 to 1.54)	73.8 (70.7 to 76.7)	73.1 (69.1 to 76.8)	−0.04 (−0.69 to 0.62)
Missouri	70.7 (65.4 to 75.4)	63.6 (57.4 to 69.3)	−0.14 (−1.32 to 1.05)	77.2 (73.7 to 80.4)	75.0 (72.0 to 77.8)	−0.05 (−0.70 to 0.61)
Montana	65.5 (59.6 to 70.9)	53.7 (48.1 to 59.3)	−0.81 (−2.96 to 1.39)	77.8 (74.2 to 81.0)	74.8 (72.0 to 77.5)	−0.11 (−0.40 to 0.18)
Nebraska	72.5 (67.8 to 76.7)	55.2 (49.3 to 60.9)	−0.65 (−1.66 to 0.37)	78.5 (75.4 to 81.3)	76.8 (74.1 to 79.3)	−0.26 (−0.73 to 0.22)
Nevada	65.1 (57.6 to 71.9)	49.1 (38.3 to 60.0)	−0.53 (−4.32 to 3.41)	78.8 (73.4 to 83.3)	70.5 (65.1 to 75.4)	−0.61 (−1.31 to 0.08)
New Hampshire	77.7 (73.9 to 81.0)	65.3 (57.7 to 72.1)	−0.98 (−2.42 to 0.48)	84.9 (82.3 to 87.3)	80.8 (78.4 to 83.0)	−0.37 (−0.73 to 0.00)
New Jersey	75.5 (69.4 to 80.7)	66.2 (60.4 to 71.5)	−0.93 (−2.03 to 0.19)	80.7 (76.1 to 84.5)	76.3 (73.0 to 79.2)	−0.14 (−0.80 to 0.51)
New Mexico	59.5 (54.8 to 64.0)	44.7 (37.6 to 51.9)	−1.59 (−2.37 to −0.81)	76.8 (73.7 to 79.6)	69.4 (65.6 to 73.0)	−0.08 (−0.83 to 0.67)
New York	73.5 (68.9 to 77.6)	66.4 (62.2 to 70.3)	−0.69 (−1.61 to 0.24)	85.5 (82.5 to 88.1)	79.2 (77.0 to 81.3)	−0.11 (−0.61 to 0.41)
North Carolina	77.0 (72.5 to 81.0)	62.8 (55.9 to 69.2)	−0.52 (−1.23 to 0.20)	85.1 (82.2 to 87.6)	79.1 (74.9 to 82.7)	−0.19 (−0.51 to 0.12)
North Dakota	71.1 (65.8 to 75.8)	63.3 (55.8 to 70.2)	−0.27 (−2.29 to 1.79)	81.2 (77.6 to 84.3)	80.2 (77.1 to 83.1)	0.10 (−0.50 to 0.69)
Ohio	69.8 (64.7 to 74.3)	57.2 (53.0 to 61.3)	−0.76 (−1.97 to 0.47)	81.2 (77.9 to 84.1)	75.6 (73.5 to 77.6)	−0.31 (−0.68 to 0.05)
Oklahoma	65.4 (61.5 to 69.1)	53.5 (47.9 to 58.9)	−0.35 (−1.14 to 0.44)	73.6 (71.1 to 75.9)	69.3 (66.2 to 72.3)	−0.14 (−0.76 to 0.48)
Oregon	64.8 (59.1 to 70.0)	54.6 (49.2 to 59.9)	−0.75 (−1.68 to 0.19)	81.4 (77.9 to 84.4)	78.0 (74.9 to 80.9)	0.13 (−0.71 to 0.98)
Pennsylvania	72.1 (69.1 to 74.8)	66.3 (58.4 to 73.4)	−0.19 (−0.85 to 0.48)	81.4 (79.4 to 83.2)	75.7 (71.1 to 79.7)	−0.18 (−0.93 to 0.59)
Rhode Island	79.8 (75.6 to 83.4)	68.7 (61.9 to 74.8)	−0.92 (−1.93 to 0.11)	90.7 (88.2 to 92.7)	86.0 (83.6 to 88.1)	−0.20 (−0.48 to 0.09)
South Carolina	67.7 (62.3 to 72.6)	60.5 (55.2 to 65.6)	−0.43 (−1.63 to 0.78)	80.1 (76.9 to 83.0)	79.7 (77.6 to 81.6)	0.36 (−0.37 to 1.10)
South Dakota	67.6 (63.1 to 71.9)	77.7 (67.7 to 85.3)	1.37 (0.12 to 2.63)	81.2 (78.5 to 83.7)	72.8 (64.6 to 79.7)	−0.17 (−0.70 to 0.36)
Tennessee	72.2 (66.5 to 77.4)	60.8 (54.0 to 67.2)	−0.98 (−2.38 to 0.45)	79.8 (76.2 to 82.9)	75.1 (71.6 to 78.3)	−0.21 (−0.62 to 0.21)
Texas	59.4 (55.3 to 63.3)	54.4 (48.5 to 60.2)	−0.11 (−1.18 to 0.96)	76.7 (73.7 to 79.6)	73.8 (70.6 to 76.7)	−0.03 (−0.61 to 0.56)
Utah	58.8 (52.3 to 65.1)	53.0 (48.7 to 57.3)	−1.26 (−3.46 to 1.00)	79.4 (75.3 to 82.9)	74.6 (71.9 to 77.1)	−0.31 (−1.48 to 0.88)
Vermont	69.3 (65.1 to 73.3)	49.3 (44.3 to 54.2)	−1.75 (−2.32 to −1.17)	84.1 (81.3 to 86.6)	75.6 (73.1 to 78.0)	−0.47 (−0.85 to −0.08)
Virginia	70.2 (64.2 to 75.5)	62.7 (57.7 to 67.4)	−0.06 (−1.04 to 0.92)	79.4 (75.4 to 82.9)	77.4 (74.9 to 79.7)	−0.13 (−0.55 to 0.30)
Washington	65.7 (61.0 to 70.1)	50.6 (47.8 to 53.5)	−0.64 (−1.59 to 0.31)	81.6 (78.5 to 84.3)	75.4 (73.9 to 76.8)	−0.22 (−0.49 to 0.06)
West Virginia	71.6 (66.2 to 76.4)	60.4 (54.4 to 66.1)	−0.26 (−1.43 to 0.92)	77.9 (74.6 to 80.9)	76.2 (73.3 to 78.8)	−0.06 (−0.74 to 0.61)
Wisconsin	75.9 (71.4 to 79.8)	58.3 (53.5 to 62.9)	−0.69 (−2.28 to 0.92)	85.1 (82.3 to 87.6)	82.1 (80.1 to 83.9)	−0.36 (−0.97 to 0.25)
Wyoming	60.8 (55.6 to 65.7)	50.6 (43.5 to 57.8)	0.17 (−1.18 to 1.55)	73.6 (70.1 to 76.8)	65.2 (61.7 to 68.4)	−0.41 (−1.44 to 0.64)

Abbreviations: ABPC, average biennial percentage change; BRFSS, Behavioral Risk Factor Surveillance System.

^a^ The mammography use prevalence reported was weighted screening prevalence adjusted for the complex survey design.

^b^ The ABPC was calculated as a geometric weighted average of the biennial percentage changes of various segments of mammography use prevalence trends in the US from 2002 to 2022. The estimates were adjusted for the methodological changes in the BRFSS survey method in 2011.

### Mammography Use Following the 2009 USPSTF Recommendations

**Figure 2.**
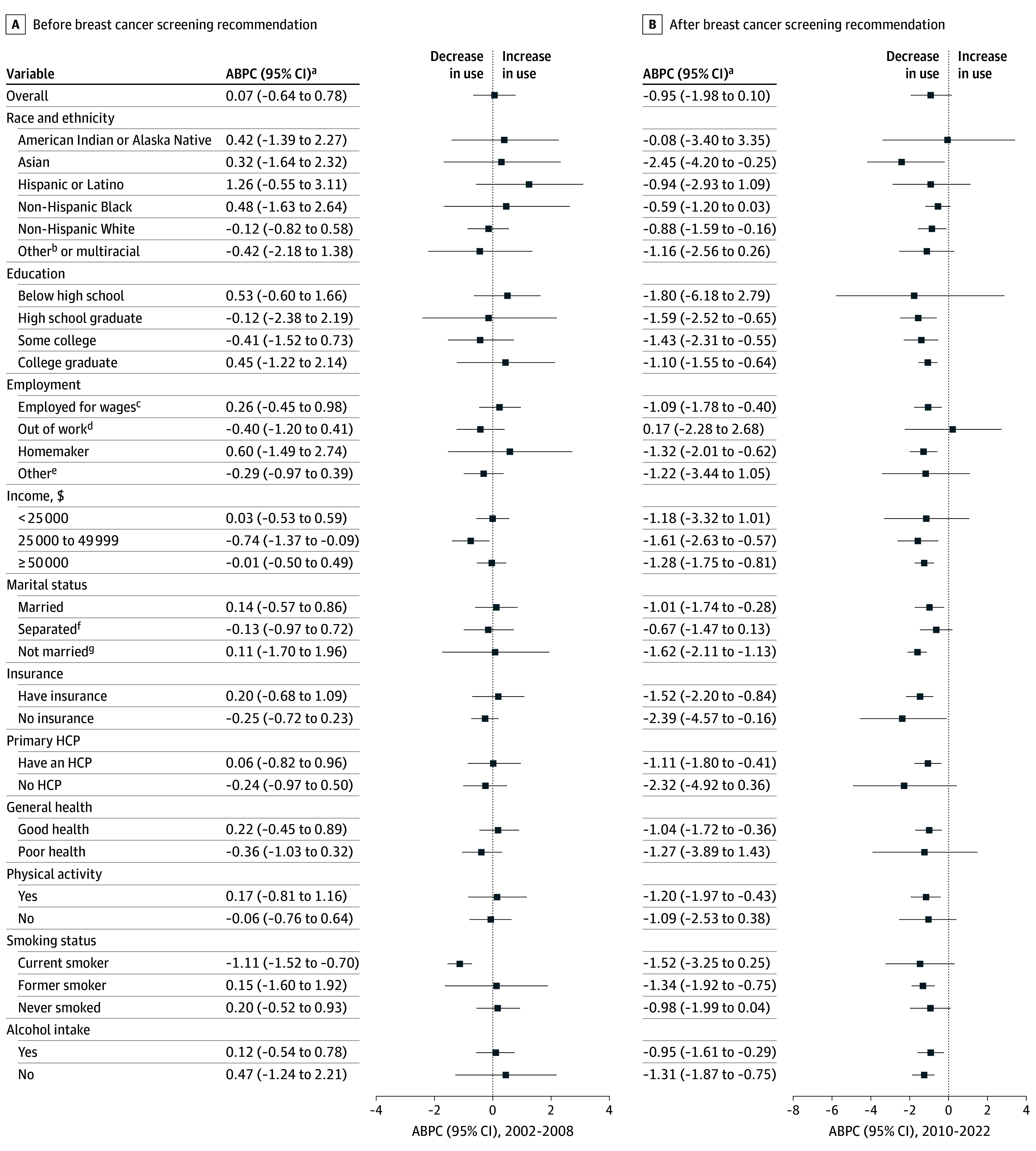
Forest Plots of Biennial Changes in Mammography Use Among Women Aged 40 to 49 Years Before and After the 2009 US Preventive Services Task Force (USPSTF) Breast Cancer Screening Recommendation Forest plots show mammography use before (2002-2008; A) and after (2010-2022; B) the USPSTF recommendation. ABPC indicates average biennial percentage change; HCP, health care practitioner. ^a^The ABPC was calculated as a geometric weighted average of the biennial percentage changes in mammography use prevalence trends. The estimates were adjusted for the methodological changes in the Behavioral Risk Factor Surveillance System (BRFSS) survey methods in 2011. ^b^Other races included Native Hawaiian or Other Pacific Islander and races and ethnicities not specified and termed as *Others* in the BRFSS dataset. ^c^Employed for wages includes the employed for wages and the self-employed. ^d^Out of work group included women who were out for work, both less than 1 year and more than 1 year. ^e^Other employment groups include students, the retired, and women unable to work. ^f^Separated includes divorced, widowed, and separated women. ^g^Not married includes never married and a member of an unmarried couple.

Following the 2009 USPSTF recommendation, the prevalence of mammography use among women aged 40 to 49 years decreased from 68.8% (95% CI, 68.0% to 69.6%) in 2010 to 59.2% (95% CI, 57.9% to 60.4%) in 2022 (eTable 3 in Supplement 1). However, the biennial change was not statistically significant (−0.95%; 95% CI, −1.98% to 0.10%). Significant reductions were observed among non-Hispanic White (−0.88%; 95% CI, −1.59% to −0.16%) and Asian women (−2.45%; 95% CI, −4.20% to −0.25%), whereas there was no significant reduction among non-Hispanic Black women (−0.59%; 95% CI, −1.20% to 0.03%) from 2010 to 2022 (Figure 2). A significant decline occurred among uninsured women (−2.39%; 95% CI, −4.57% to −0.16%) from 2010 to 2022. Women with a health care practitioner also experienced a significant decline in mammography use during this period (−1.11%; 95% CI, −1.80% to −0.41%).

**Figure 3.**
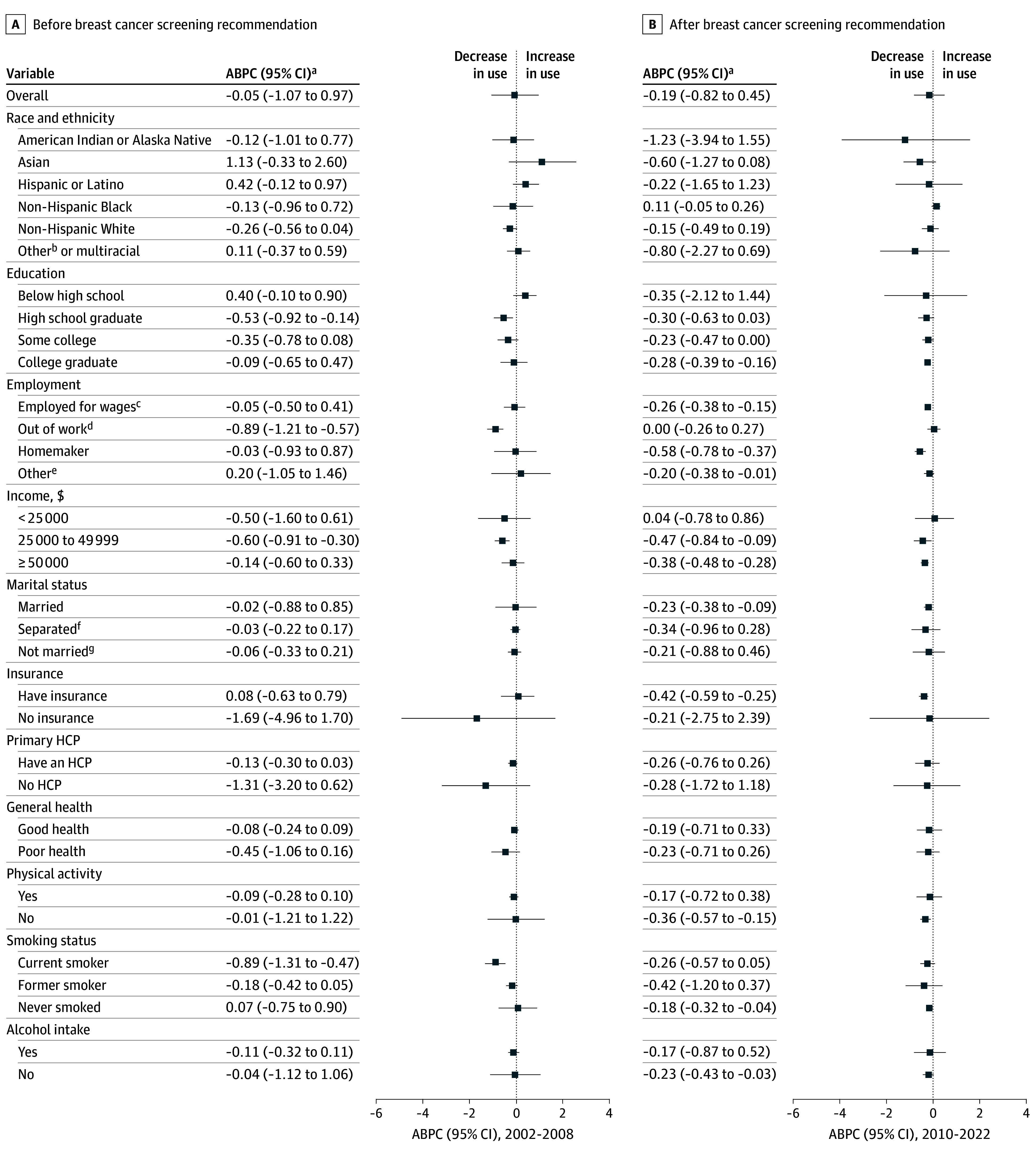
Forest Plots of Biennial Changes in Mammography Use Among Women Aged 50 to 74 Years Before and After the 2009 US Preventive Services Task Force (USPSTF) Breast Cancer Screening Recommendation Forest plots show mammography use before (2002-2008; A) and after (2010-2022; B) the USPSTF recommendation. ABPC indicates average biennial percentage change; HCP, health care practitioner. ^a^The ABPC was calculated as a geometric weighted average of the biennial percent changes in mammography use prevalence trends. The estimates were adjusted for the methodological changes in the Behavioral Risk Factor Surveillance System (BRFSS) survey methods in 2011. ^b^Other races included Native Hawaiian or Other Pacific Islander and races and ethnicities not specified and termed as *Others* in the BRFSS dataset. ^c^Employed for wages includes the employed for wages and the self-employed. ^d^Out of work group included women who were out for work, both less than 1 year and more than 1 year. ^e^Other employment groups include students, the retired, and women unable to work. ^f^Separated includes divorced, widowed, and separated women. ^g^Not married includes never married and a member of an unmarried couple.

Mammography use among women aged 40 to 49 years showed a significant reduction in 17 states and Washington, DC, from 2010 to 2022. During this period, the largest reductions occurred in Vermont (−3.15%; 95% CI, −3.86% to −2.43%), New Mexico (−2.74%; 95% CI, −3.70% to −1.77%), California (−2.47%; 95% CI, −4.33% to −0.57%), Massachusetts (−1.99%; 95% CI, −2.615 to −1.37%), New Jersey (−1.91%; 95% CI, −3.49% to −0.30%), and Maine (−1.86%; 95% CI, −2.32% to −1.39%) (eTable 7 in Supplement 1). In contrast, only South Dakota (2.17%; 95% CI, 0.63% to 3.73%) and Mississippi (1.27%; 95% CI, 0.36% to 2.19%) experienced significant increases. Although the USPSTF recommendation for women aged 50 to 74 years remained unchanged, mammography use in this group declined from 79.7% (95% CI, 79.4% to 80.1%) in 2010 to 77.0% (95% CI, 76.4% to 77.6%) in 2022. Significant declines were observed among homemakers, insured women, and higher-income women during this period (Figure 3). State-level trends for this age group are shown in eTable 7 in Supplement 1.

### COVID-19 Pandemic and Mammography Use

During the COVID-19 pandemic, there were not statistically significant decreases in mammography use among women aged 40 to 49 years (−0.15%; 95% CI, −1.27% to 0.97%) and 50 to 74 years (−0.16%; 95% CI, −0.48% to 0.17%) (eTable 8 in Supplement 1). However, significant pandemic-associated declines were observed among uninsured women aged 40 to 49 years (−4.08%; 95% CI, −6.29% to −1.86%) and among American Indian or Alaska Native (−2.27%; 95% CI, −2.43% to −2.11%) and lower-income (−0.96%; 95% CI, −1.47% to −0.46%) women aged 50 to 74 years. At the state level, 20 states experienced reduced mammography use among women aged 40 to 49 years, with significant reductions in 3 states (eTable 9 in Supplement 1). Among women aged 50 to 74 years, 27 states showed downward trends, with a significant reduction only in Hawaii.

## Discussion

This cross-sectional study provides a comprehensive assessment of 20-year trends in mammography use among US women aged 40 to 49 and 50 to 74 years. Although overall mammography prevalence in both age groups was lower in 2022 than in 2002, these declines were not statistically significant at the population level. In contrast, following the 2009 USPSTF recommendation, mammography use declined significantly among women aged 40 to 49 years who were non-Hispanic White, Asian, uninsured, and women with a usual health care practitioner, whereas there was no significant decline among non-Hispanic Black women. Trends varied by socioeconomic, behavioral, and geographic strata across both age groups, indicating substantial heterogeneity in mammography use over time.

This study assessed whether USPSTF breast cancer screening guideline changes were associated with differential trends in mammography use across population subgroups. Because USPSTF recommendations are disseminated through multiple channels—including clinical practice, professional organizations, and public media—we hypothesized that guideline changes would not be associated with mammography use uniformly across women. Subgroup analyses by health care access, socioeconomic status, and behavioral factors, such as smoking and alcohol use, were used to characterize heterogeneity in mammography trends and to capture differences in health behavior profiles, risk perception, and potential responsiveness to evolving screening guidance.

Following the 2009 USPSTF recommendation against routine screening for women aged 40 to 49 years,^6^ mammography use reduced significantly among non-Hispanic White, Asian, and uninsured women, whereas no significant decline occurred among non-Hispanic Black women. This reduction was consistent with the 2009 USPSTF recommendation, which was informed by emerging evidence on the balance of benefits and harms of screening in younger women and emphasized individualized decision-making rather than routine population-wide screening. Observed declines among younger women may, therefore, represent guideline-concordant behavior, particularly among women who are more engaged with the health care system. The observed racial difference in mammography use may reflect both differential responsiveness to guideline changes^24^ and the impact of community outreach or screening programs that support low-income and uninsured or underinsured non-Hispanic Black women.^25^ Non-Hispanic Black women in the US experience substantially higher breast cancer mortality compared with non-Hispanic White women,^26^ despite higher mammography participation.^27^ This persistent mortality gap underscores the need for targeted screening outreach and community programs to improve coverage and continuity among non-Hispanic Black women. National data from the American Cancer Society showed that in 2023, 75% of non-Hispanic Black women aged 45 years or older were up to date with mammography, compared with 69% of non-Hispanic White, 64% of Hispanic, and 71% of Asian women.^28^ Similarly, the National Breast and Cervical Cancer Early Detection Program consistently reached a higher proportion of eligible non-Hispanic Black women than non-Hispanic White women in 2018 to 2019 (14.6% vs 9.8%) and 2020 to 2021 (11.5% vs 8.0%).^25^ These patterns suggest that targeted outreach and safety-net programs may have buffered declines in screening participation among non-Hispanic Black women, even as declines persisted in other populations.

Direct comparisons of long-term, nationwide trends are limited because of the scarcity of continuous data. One prior study^13^ reported significant declines in mammography use among non-Hispanic Black women following the 2009 USPSTF recommendation. However, it did not account for the 2011 BRFSS methodological changes,^14^ which substantially affected prevalence estimates and varied by race. Trends after the methodological update also differed by race: non-Hispanic White women exhibited a continuous decline, whereas non-Hispanic Black women experienced an initial decrease in 2012, followed by an increase in 2014. By incorporating the 2011 methodological adjustment, our study provides robust temporal estimates, showing significant reductions among White but not Black women aged 40 to 49 years following the 2009 USPSTF recommendation.

Few studies have reported breast cancer screening trends among women aged 50 to 74 years, either in a specific state^29^ or nationally,^30^ and none has comprehensively assessed long-term mammography use across all states while stratifying by sociodemographic, behavioral, and health care access factors. Our findings address this gap, revealing that even in this age group—targeted for biennial screening—mammography use showed a downward trend following the 2009 USPSTF recommendation, with a significant reduction among insured, homemakers, and higher-income women. These patterns suggest that changes in national guidelines can subtly yet meaningfully influence preventive care uptake, even among populations for whom recommendations remain unchanged.

Health care access remains an important determinant of mammography use among US women^27,31^; however, our findings suggest a more nuanced association between access and mammography trends following guideline changes. Among women aged 40 to 49 years, significant declines in mammography use were observed among insured women and those with a regular health care practitioner. However, women without a regular health care practitioner consistently exhibited substantially lower mammography prevalence from 2002 to 2022, and the lack of a statistically significant decline in this group likely reflects the low uptake in this group. At the same time, the largest reductions occurred among uninsured women, reflecting persistent financial and structural barriers. These patterns align with evidence that insurance coverage reduces cost-associated barriers to screening,^32,33^ while a regular health care practitioner supports care continuity, trust, and adherence to guideline-based clinical decision-making. Collectively, these findings suggest that declines in mammography use reflect both structural constraints and increased responsiveness to guideline-driven changes in screening practice.

Geographic variation in mammography use was pronounced across both age groups, with consistently lower utilization observed in the Western US compared with the Eastern US. Although most states exhibited downward trends, statistically significant declines in both age groups were limited to only Vermont, Massachusetts, Kansas, and Washington DC, whereas Mississippi showed a significant increase among younger women. Interestingly, some states with declines—such as Vermont, Massachusetts, and Washington, DC—had expanded Medicaid and rank among the nation’s highest-performing health systems.^34^ In contrast, Kansas and Mississippi did not adopt Medicaid expansion and rank low in health system performance, with Mississippi the lowest nationwide.^34^ These patterns suggest that reductions in mammography use are not solely explained by insurance coverage or overall system quality. Instead, evolving guidelines, population-level attitudes toward screening, and state-specific health care delivery models are likely associated with the observed patterns.^35^ This also highlights that even high-performing states can experience declines in preventive service utilization.

These findings can be interpreted within the context of increasing breast cancer incidence among women younger than 50 years in the US. Declines in screening participation may delay diagnosis and shift cancers toward more advanced stages at detection. The observed reductions in mammography use, therefore, raise important public health considerations, particularly as younger women experience rising disease burden alongside ongoing debates about the balance of screening benefits and harms. Our findings underscore the need for clear risk-based screening communication and targeted strategies to guideline-concordant decision-making to ensure timely detection among younger women who may be at elevated risk, even as screening guidelines continue to evolve.

### Limitations and Strengths

This study has limitations. First, mammography use was self-reported, which may introduce recall and social desirability bias; however, self-report remains the standard approach in large-scale surveillance and has been shown to yield reasonably valid estimates. Second, BRFSS does not distinguish between screening and diagnostic mammography, nor does it capture cancer stage, tumor characteristics, or outcomes, which preclude direct assessment of whether declines in mammography use were associated with increased advanced-stage disease or mortality. Future studies linking population-based screening data with cancer registries are needed to assess the long-term clinical consequences of changes in mammography use. Third, the cross-sectional design limits causal inference at the individual level. Fourth, although the survey captures key sociodemographic and health care access variables, it lacks data on individual medical history, practitioner recommendations, and facility-level availability, which may influence screening behavior. Fifth, biennial measurement may underestimate transient pandemic-related declines, and joinpoint estimates are sensitive to the selected time frame; trends should be interpreted within this context.

Despite these limitations, the study has important strengths. It draws on 2 decades of nationally representative data with consistent methods, allowing a robust assessment of long-term trends. The large sample size provided sufficient power to detect subgroup and state-level differences, providing a granular understanding of disparities in mammography use. By integrating national and geographic perspectives, the analysis captures heterogeneity often masked in aggregate estimates. Contextualizing findings within evolving screening guidelines and the COVID-19 pandemic further enhances the relevance of these insights for understanding dynamics in US mammography use.

## Conclusions

In this cross-sectional study of 2 619 292 US women, mammography use did not significantly decline in the overall population over the past 2 decades. However, significant reductions were observed across sociodemographic, health care access, and geographic subgroups of women aged 40 to 49 years following the 2009 USPSTF recommendation. Our findings underscore the need for clear, risk-based screening communication and targeted strategies are needed to support guideline-concordant decision-making, ensure timely detection among younger women who may be at elevated risk, and promote equitable access to breast cancer screening.

## Notes

Supplement 1. eTable 1. BRFSS survey questions and response options used to define study variables, 2002–2022 eTable 2. Summary statistics, 2002–2022 eTable 3. Prevalence of mammography use among women aged 40–49 years in the US, 2002, 2008, 2010, 2018, and 2022 eTable 4. Prevalence of mammography use among women aged 50–74 years in the US, 2002, 2008, 2010, 2018, and 2022 eTable 5. Prevalence of mammography use among US women aged 40–49 years by states, 2002, 2008, 2010, 2018, and 2022 eTable 6. Prevalence of mammography use among US women aged 50–74 years by states, 2002, 2008, 2010, 2018, and 2022 eTable 7. Biennial changes in mammography use by states before and after the 2009 USPSTF breast cancer screening recommendation eTable 8. Differences in biennial changes in mammography use between 2012–2022 and 2012–2018 eTable 9. Differences in biennial changes in mammography use by state between 2012–2022 and 2012–2018 eFigure 1. Mammography use prevalence among women aged 40–49 years in 2002 and 2022 eFigure 2. Mammography use prevalence among women aged 50–74 years in 2002 and 2022 eFigure 3. Bivariate spatial patterns of mammography use in 2002 and 2022 among women aged 40–49 and 50–74 years eFigure 4. Average biennial percent changes in mammography use among women aged 40–49 and 50–74 years, 2002–2022

Supplement 2. Data Sharing Statement
