# Supplementary material for: Trends in Mammography Use Among Women Aged 40 to 74 Years in the US, 2002-2022
Source: JAMA Netw Open. 2026 Mar 26;9(3):e263529. doi: 10.1001/jamanetworkopen.2026.3529 (PMC13022741; doi:10.1001/jamanetworkopen.2026.3529)
Supplement: Supplement 1. — eTable 1. BRFSS survey questions and response options used to define study variables, 2002–2022 eTable 2. Summary statistics, 2002–2022 eTable 3. Prevalence of mammography use among women aged 40–49 years in the US, 2002, 2008, 2010, 2018, and 2022 eTable 4. Prevalence of mammography use among women aged 50–74 years in the US, 2002, 2008, 2010, 2018, and 2022 eTable 5. Prevalence of mammography use among US women aged 40–49 years by states, 2002, 2008, 2010, 2018, and 2022 eTable 6. Prevalence of mammography use among US women aged 50–74 years by states, 2002, 2008, 2010, 2018, and 2022 eTable 7. Biennial changes in mammography use by states before and after the 2009 USPSTF breast cancer screening recommendation eTable 8. Differences in biennial changes in mammography use between 2012–2022 and 2012–2018 eTable 9. Differences in biennial changes in mammography use by state between 2012–2022 and 2012–2018 eFigure 1. Mammography use prevalence among women aged 40–49 years in 2002 and 2022 eFigure 2. Mammography use prevalence among women aged 50–74 years in 2002 and 2022 eFigure 3. Bivariate spatial patterns of mammography use in 2002 and 2022 among women aged 40–49 and 50–74 years eFigure 4. Average biennial percent changes in mammography use among women aged 40–49 and 50–74 years, 2002–2022 [file jamanetwopen-e263529-s001.pdf]

Supplemental Online Content

Al Hasan SM, Bennett DL, Toriola AT. Trends in mammography use among women aged 40 to 74 years in the US, 2002–2022. *JAMA Netw Open*. 2026;9(3):e263529. doi:10.1001/jamanetworkopen.2026.3529

eTable 1. BRFSS survey questions and response options used to define study variables, 2002–2022

eTable 2. Summary statistics, 2002–2022

eTable 3. Prevalence of mammography use among women aged 40–49 years in the US, 2002, 2008, 2010, 2018, and 2022

eTable 4. Prevalence of mammography use among women aged 50–74 years in the US, 2002, 2008, 2010, 2018, and 2022

eTable 5. Prevalence of mammography use among US women aged 40–49 years by states, 2002, 2008, 2010, 2018, and 2022

eTable 6. Prevalence of mammography use among US women aged 50–74 years by states, 2002, 2008, 2010, 2018, and 2022

eTable 7. Biennial changes in mammography use by states before and after the 2009 USPSTF breast cancer screening recommendation

eTable 8. Differences in biennial changes in mammography use between 2012–2022 and 2012–2018

eTable 9. Differences in biennial changes in mammography use by state between 2012–2022 and 2012–2018

eFigure 1. Mammography use prevalence among women aged 40–49 years in 2002 and 2022

eFigure 2. Mammography use prevalence among women aged 50–74 years in 2002 and 2022

eFigure 3. Bivariate spatial patterns of mammography use in 2002 and 2022 among women aged 40–49 and 50–74 years

eFigure 4. Average biennial percent changes in mammography use among women aged 40–49 and 50–74 years, 2002–2022

This supplemental material has been provided by the authors to give readers additional information about their work.

© 2026 Al Hasan SM et al. *JAMA Network Open*.

eTable 1. BRFSS survey questions and response options used to define study variables, 2002–2022

Variable domain	Variable	BRFSS Question	Responses
**Mammography**	**HADMAM**	**Have you ever had a mammogram?**	Yes
			No
			Don’t know/Not sure
			Refused
	**HOWLONG**	**How long has it been since you had your last mammogram?**	Within the past year (anytime <12 months ago)
			Within the past 2 years (1 year but <2 years ago)
			Within the past 3 years (2 years but <3 years age)
			Within the past 5 years (3 years but < 5 years ago)
			5 or more years ago
			Do not know/Not sure
			Refused
**Sociodemographic**	**Race**	**Race/ethnicity categories**	White only, non-Hispanic
			Black only, non-Hispanic
			American Indian or Alaska Native only, non-Hispanic
			Asian only, non-Hispanic
			Native Hawaiian or Other Pacific Islander only, non-Hispanic
			Other race only, non-Hispanic
			Multiracial, non-Hispanic
			Hispanic
			Do not know/Not sure/Refused
	**Education**	**Level of education completed**	Did not graduate from high school
			Graduate high school
			Attended college or technical school
			Graduated from a college or technical school
			Do not know/Not sure
	**Employment**	**Are you currently…?**	Employed for wages
			Self-employed
			Out of work for 1 year or more
			Out of work for < 1 year
			A homemaker

© 2026 Al Hasan SM et al. *JAMA Network Open*.

			A student
			Retired
			Unable to work
			Refused
	**Income**	**Income categories**	Less than $15,000
			$15,000 to < $25,000
			$25,000 to < $35,000
			$35,000 to < $50,000
			$50,000 to < $100,000
			$100,000 to < $200,000
			$200,000 or more
			Do not know/Not sure
	**Marital status**	**Are you: (marital status)**	Married
			Divorced
			Widowed
			Separated
			Never married
			A member of an unmarried couple
			Refused
**Health care access**	**Health insurance**	**Adults who had some form of health insurance**[a]	Have some form of insurance
			Do not have some form of health insurance
			Do not know/Not sure/Refused
	**Primary HCP**	**Do you have one person that you think of as your HCP?**[b]	Yes, only one
			More than one
			No
			Do not know/Not sure
			Refused
**Behavioral**	**General health**	**Adults with good or better health**[c]	Good or better health
			Fair or poor health
			Do not know/Not sure/Refused
	**Physical activity**	**Doing physical activity or exercise during the past 30 days**[d]	Had physical activity or exercise

© 2026 Al Hasan SM et al. *JAMA Network Open*.

		No physical activity or exercise in the last 30 days
		Do not know/Refused
**Smoking status**	**Four-level smoker status: Everyday, Someday, Former, No**	Current smoker (now smokes every day)
		Current smoker (now smokes some days)
		Former smoker
		Never smoker
		Do not know/Refused
**Alcohol intake**	**Having had at least one drink of alcohol in the past 30 days.**	Yes
		No
		Do not know/Not sure
		Refused

[a]This variable is calculated from the question, what is the current primary source of your health insurance? The variable name of this question is PRIMINSR. The responses for this variable are: A plan purchased through an employer or union (including plans purchased through another person´s employer), A private nongovernmental plan that you or another family member buys on your own, Medicare, Medigap, Medicaid, Children´s Health Insurance Program (CHIP), Military related health care: TRICARE (CHAMPUS) / VA health care / CHAMP-VA, Indian Health Service, State sponsored health plan, Other government program, No coverage of any type, Do not know/Not sure, Refused.

[b]HCP stands for health care provider. The detailed question is: Do you have one person (or a group of doctors) that you think of as your personal health care provider?

[c]This variable is calculated from the following question: Would you say that in general your health is: The variable name of this question is GENHELTH. The responses for this variable are: Excellent, very good, good, fair, poor, Do not know/Not sure, Refused.

[d]The detailed and exact wording question for this is: Adults who reported doing physical activity or exercise during the past 30 days other than their regular job.

© 2026 Al Hasan SM et al. *JAMA Network Open*.

eTable 2: Summary statistics, 2002–2022

	Total	Women information					Weighted Prevalence, % (95% CI)	
**BRFSS Study Cycle**	**Total respondents**	**Surveyed**	**Responded**	**Missing response**	**40–49 years**	**50–74 years**	**40–49 years**	**50–74 years**
**BRFSS 2002**	247,964	148,702	146,624	2,078	29,883	52,176	69.9 (68.9–70.8)	81.3 (80.6–81.9)
**BRFSS 2004**	303,822	186,256	184,923	1,333	36,681	71,958	66.4 (65.5–67.4)	80.3 (79.7–80.9)
**BRFSS 2006**	355,710	220,302	215,586	4,716	40,488	93,178	69.2 (68.3–70.1)	81.6 (81.1–82.1)
**BRFSS 2008**	414,509	258,806	254,828	3,978	44,063	122,374	69.9 (69.1–70.6)	81.2 (80.8–81.6)
**BRFSS 2010**	451,075	280,961	275,051	5,910	43,183	142,332	68.8 (68–69.6)	79.7 (79.4–80.1)
**BRFSS 2012**	475,687	283,950	276,090	7,860	39,601	139,761	65.8 (64.9–66.8)	78.7 (78.3–79.2)
**BRFSS 2014**	464,664	271,694	256,342	15,352	34,943	137,916	65.5 (64.5–66.4)	78.5 (78.1–79)
**BRFSS 2016**	486,303	275,650	261,007	14,643	33,170	140,000	62.5 (61.4–63.6)	78.5 (78–78.9)
**BRFSS 2018**	437,436	239,051	227,057	11,994	29,081	116,756	62.2 (61–63.4)	78.9 (78.3–79.5)
**BRFSS 2020**	401,958	218,027	203,520	14,507	27,999	102,774	58.3 (56.9–59.7)	78.5 (77.8–79.2)
**BRFSS 2022**	445,132	235,893	221,813	14,080	30,547	110,477	59.2 (57.9–60.4)	77 (76.4–77.6)
**Total**	4,484,260	2,619,292	2,522,841	96,451				

Notes: In the 2016 BRFSS, 19 respondents originally coded as "Refused" (total refused: 66) for gender were reclassified as female because they answered questions about mammography, thereby increasing the sample size from 275,631 to 275,650. In 2018, 140 respondents with "Refused" (total refused: 682, and "Do not know": 431) gender responses were similarly reclassified, increasing the sample from 238,911 to 239,051. In 2020, two gender variables were present. We used @_SEX (n = 218,027) as the primary source; discrepancies with SEXVAR (e.g., 196 cases coded as female in @_SEX but male in SEXVAR) were resolved by retaining the @_SEX coding.

© 2026 Al Hasan SM et al. *JAMA Network Open*.

eTable 3: Prevalence of mammography use among women aged 40–49 years in the US, 2002, 2008, 2010, 2018, and 2022

	40–49 years, % (95% CI)[a]				
**Characteristics**	**BRFSS 2002**	**BRFSS 2008**	**BRFSS 2010**	**BRFSS 2018**	**BRFSS 2022**
**Overall**	69.9 (68.9 to 70.8)	69.9 (69.1 to 70.6)	68.8 (68.0 to 69.6)	62.2 (61.0 to 63.4)	59.2 (57.9 to 60.4)
**Race and ethnicity**					
American Indian/Alaska Native	63.2 (54.1 to 71.4)	66.1 (58.4 to 73.1)	62.9 (53.0 to 71.7)	48.3 (39.7 to 57.1)	55.2 (45.8 to 64.2)
Asian	60.4 (50.0 to 69.9)	66.9 (61.1 to 72.3)	63.8 (58.4 to 68.9)	52.0 (44.3 to 59.5)	55.4 (48.4 to 62.3)
Hispanic or Latino	63.1 (58.9 to 67.2)	67.2 (64.3 to 70.0)	69.6 (67.2 to 71.9)	63.3 (59.9 to 66.7)	55.1 (51.6 to 58.5)
Non-Hispanic Black	72.3 (69.3 to 75.1)	73.4 (70.8 to 75.7)	70.7 (68.2 to 73.2)	67.8 (64.3 to 71.0)	64.9 (61.8 to 67.9)
Non-Hispanic White	71.3 (70.3 to 72.2)	70.2 (69.4 to 71.0)	69.0 (68.1 to 69.9)	62.1 (60.9 to 63.4)	60.4 (59.0 to 61.7)
Other[b] or multiracial	66.2 (59.6 to 72.2)	65.2 (59.2 to 70.8)	57.4 (52.0 to 62.7)	57.0 (50.5 to 63.3)	54.7 (48.2 to 61.0)
**Education**					
Below high school	56.3 (51.7 to 60.9)	59.9 (56.4 to 63.3)	57.5 (54.2 to 60.8)	55.0 (50.8 to 59.2)	45.1 (40.0 to 50.2)
High school graduate	66.7 (65.0 to 68.3)	65.4 (63.9 to 66.9)	63.3 (61.7 to 64.9)	59.1 (56.2 to 61.9)	56.1 (53.2 to 58.9)
Some college	71.7 (70.0 to 73.4)	68.2 (66.7 to 69.7)	68.0 (66.5 to 69.4)	60.5 (58.2 to 62.7)	56.0 (53.7 to 58.2)
College graduate	75.5 (74.0 to 77.0)	76.1 (75.0 to 77.2)	74.5 (73.4 to 75.6)	67.6 (65.8 to 69.2)	67.1 (65.6 to 68.6)
**Employment**					
Employed for wages[c]	71.8 (70.7 to 72.9)	72.1 (71.2 to 72.9)	71.5 (70.6 to 72.4)	64.4 (63.0 to 65.8)	60.6 (59.2 to 62.1)
Out of work[d]	60.7 (56.2 to 65.1)	57.6 (54.0 to 61.1)	60.4 (57.5 to 63.3)	49.1 (43.0 to 55.1)	56.6 (51.6 to 61.5)
Homemaker	64.5 (61.6 to 67.2)	66.2 (64.0 to 68.3)	64.8 (62.6 to 66.9)	58.4 (55.1 to 61.5)	55.2 (51.2 to 59.1)
Others[e]	67.3 (63.0 to 71.3)	65.5 (62.7 to 68.2)	63.3 (60.7 to 65.9)	60.0 (56.4 to 63.4)	53.5 (49.5 to 57.4)
**Income**					
Less than $25,000	58.0 (55.5 to 60.4)	56.5 (54.4 to 58.5)	56.9 (55.0 to 58.8)	56.1 (53.2 to 58.9)	49.6 (46.5 to 52.7)
$25,000 to $49,999	68.2 (66.4 to 70.0)	65.0 (63.2 to 66.7)	62.7 (60.8 to 64.6)	56.9 (54.0 to 59.7)	53.4 (50.4 to 56.3)
More than $50,000	76.9 (75.5 to 78.2)	76.1 (75.2 to 77.1)	75.4 (74.4 to 76.4)	66.8 (65.2 to 68.3)	63.7 (62.1 to 65.3)
**Marital status**					
Married	72.2 (71.0 to 73.3)	72.2 (71.3 to 73.1)	71.2 (70.3 to 72.1)	65.1 (63.6 to 66.6)	62.1 (60.5 to 63.7)
Separated[f]	65.6 (63.6 to 67.6)	63.9 (62.1 to 65.6)	63.9 (62.1 to 65.7)	59.3 (56.7 to 61.9)	56.1 (53.6 to 58.6)
Not married[g]	64.1 (61.1 to 67.0)	64.9 (62.6 to 67.1)	60.7 (58.4 to 62.9)	54.8 (51.9 to 57.7)	52.4 (49.8 to 55.0)
**Insurance**					
Have insurance	73.7 (72.7 to 74.6)	73.8 (73.0 to 74.6)	73.4 (72.6 to 74.1)	64.7 (63.5 to 66.0)	61.9 (60.6 to 63.1)
No insurance	46.7 (43.8 to 49.6)	45.1 (42.7 to 47.5)	43.4 (41.1 to 45.7)	46.4 (42.4 to 50.5)	33.2 (28.8 to 37.8)
**Primary HCP**					
Have an HCP	73.9 (72.9 to 74.8)	73.6 (72.8 to 74.4)	72.7 (71.9 to 73.5)	66.8 (65.5 to 68.0)	63.3 (62.0 to 64.6)
No HCP	44.9 (41.9 to 48.0)	45.3 (42.8 to 47.8)	43.2 (40.7 to 45.7)	40.3 (37.1 to 43.6)	32.5 (29.4 to 35.8)
**General health**					

© 2026 Al Hasan SM et al. *JAMA Network Open*.

Good health	70.7 (69.7 to 71.7)	71.3 (70.5 to 72.1)	70.4 (69.6 to 71.2)	63.5 (62.2 to 64.8)	60.9 (59.6 to 62.3)
Poor health	65.1 (62.3 to 67.8)	62.0 (59.8 to 64.2)	59.9 (57.7 to 62.0)	57.6 (54.7 to 60.5)	51.0 (48.0 to 54.1)
**Physical activity**					
Yes	71.8 (70.7 to 72.8)	72.1 (71.3 to 73.0)	71.1 (70.2 to 71.9)	63.7 (62.3 to 65.0)	61.1 (59.7 to 62.5)
No	64.2 (62.1 to 66.3)	63.6 (61.9 to 65.2)	61.8 (60.1 to 63.5)	57.9 (55.3 to 60.5)	53.1 (50.5 to 55.7)
**Smoking status**					
Current smoker	60.3 (58.4 to 62.2)	56.7 (55.0 to 58.5)	53.8 (51.9 to 55.7)	51.0 (48.3 to 53.7)	46.5 (43.7 to 49.4)
Former smoker	73.7 (71.6 to 75.7)	73.9 (72.3 to 75.4)	69.8 (68.1 to 71.5)	63.5 (60.8 to 66.2)	60.4 (57.8 to 63.0)
Never smoked	72.7 (71.4 to 74.0)	72.8 (71.8 to 73.8)	72.6 (71.6 to 73.5)	64.8 (63.3 to 66.3)	61.7 (60.1 to 63.3)
**Alcohol intake**					
Yes	72.9 (71.7 to 74.2)	72.3 (71.3 to 73.3)	72.0 (71.0 to 73.0)	64.4 (62.7 to 66.1)	61.8 (60.1 to 63.4)
No	66.6 (65.1 to 68.1)	67.3 (66.1 to 68.4)	65.3 (64.2 to 66.5)	59.9 (58.2 to 61.6)	56.1 (54.1 to 58.0)

[a]The prevalence of mammography use is reported as a weighted percentage, %, with 95% confidence interval (CI).

[b]Other races included Native Hawaiian/other Pacific Islander and races and ethnicities not specified and termed as “Others” in the BRFSS dataset.

[c]Employed for wages includes the employed for wages and the self-employed.

[d]Out of the work group included women who were out for work, both less than 1 year and more than 1 year.

[e]Other employment groups include students, the retired, and women unable to work.

[f]Separated includes divorced, widowed, and separated women.

[g]Not married includes never married and a member of an unmarried couple.

HCP stands for healthcare provider

© 2026 Al Hasan SM et al. *JAMA Network Open*.

eTable 4: Prevalence of mammography use among women aged 50–74 years in the US, 2002, 2008, 2010, 2018, and 2022

	**50–74 years, % (95% CI)[a]**				
**Characteristics**	**BRFSS 2002**	**BRFSS 2008**	**BRFSS 2010**	**BRFSS 2018**	**BRFSS 2022**
**Overall**	81.3 (80.6 to 81.9)	81.2 (80.8 to 81.6)	79.7 (79.4 to 80.1)	78.9 (78.3 to 79.5)	77.0 (76.4 to 77.6)
**Race and ethnicity**					
American Indian/Alaska Native	76.8 (68.4 to 83.5)	70.2 (64.9 to 75.1)	64.2 (57.8 to 70.0)	74.9 (69.7 to 79.4)	62.1 (55.8 to 68.1)
Asian	71.4 (58.4 to 81.6)	79.5 (74.4 to 83.8)	78.9 (74.9 to 82.5)	78.5 (72.5 to 83.6)	76.0 (70.5 to 80.8)
Hispanic or Latino	78.6 (74.7 to 82.1)	81.4 (79.4 to 83.2)	79.7 (78.0 to 81.2)	79.4 (76.4 to 82.0)	74.9 (72.5 to 77.1)
Non-Hispanic Black	83.1 (80.7 to 85.2)	82.2 (80.6 to 83.7)	82.4 (81.2 to 83.6)	84.1 (82.5 to 85.6)	83.3 (81.8 to 84.6)
Non-Hispanic White	81.7 (81.1 to 82.3)	81.5 (81.1 to 81.9)	79.7 (79.3 to 80.1)	78.1 (77.5 to 78.7)	77.0 (76.4 to 77.6)
Other[b] or multiracial	79.1 (73.3 to 83.9)	71.8 (67.8 to 75.4)	74.0 (71.1 to 76.7)	77.3 (73.9 to 80.3)	66.1 (60.6 to 71.3)
**Education**					
Below high school	71.4 (68.9 to 73.8)	73.5 (71.7 to 75.2)	71.5 (69.9 to 73.0)	73.3 (70.5 to 75.9)	65.6 (62.7 to 68.4)
High school graduate	80.8 (79.7 to 81.7)	79.0 (78.2 to 79.7)	76.8 (76.1 to 77.5)	77.0 (76.0 to 78.0)	74.3 (73.1 to 75.4)
Some college	82.2 (81.1 to 83.3)	81.0 (80.2 to 81.7)	79.2 (78.4 to 79.9)	78.6 (77.6 to 79.5)	77.2 (76.2 to 78.2)
College graduate	86.4 (85.1 to 87.6)	86.0 (85.3 to 86.6)	84.9 (84.3 to 85.5)	83.3 (82.4 to 84.2)	81.8 (80.9 to 82.6)
**Employment**					
Employed for wages[c]	82.1 (81.2 to 83.0)	82.0 (81.4 to 82.6)	80.7 (80.2 to 81.3)	79.2 (78.3 to 80.0)	77.4 (76.4 to 78.3)
Out of work[d]	74.6 (70.9 to 78.0)	71.2 (68.5 to 73.8)	69.5 (67.6 to 71.4)	68.7 (64.5 to 72.6)	66.9 (63.4 to 70.3)
Homemaker	79.5 (77.5 to 81.2)	78.3 (76.8 to 79.7)	77.1 (75.8 to 78.4)	75.3 (72.1 to 78.3)	71.3 (68.6 to 73.9)
Others[e]	81.5 (80.4 to 82.5)	82.2 (81.6 to 82.8)	81.0 (80.4 to 81.5)	80.2 (79.5 to 81.0)	78.0 (77.1 to 78.9)
**Income**					
Less than $25,000	73.5 (72.1 to 74.8)	72.3 (71.3 to 73.3)	70.0 (69.1 to 70.8)	72.6 (71.4 to 73.8)	67.0 (65.1 to 68.8)
$25,000 to $49,999	83.2 (82.1 to 84.3)	80.3 (79.4 to 81.1)	78.7 (77.9 to 79.5)	76.9 (75.6 to 78.2)	74.0 (72.6 to 75.3)
More than $50,000	87.5 (86.3 to 88.5)	86.6 (86.0 to 87.1)	85.8 (85.2 to 86.3)	83.0 (82.2 to 83.8)	81.3 (80.5 to 82.1)
**Marital status**					
Married	83.8 (82.9 to 84.6)	83.8 (83.3 to 84.3)	82.2 (81.8 to 82.7)	80.8 (80.0 to 81.6)	80.0 (79.3 to 80.8)
Separated[f]	77.3 (76.2 to 78.4)	76.7 (75.9 to 77.4)	75.4 (74.8 to 76.1)	76.3 (75.3 to 77.3)	72.0 (70.9 to 73.1)
Not married[g]	76.2 (73.3 to 78.9)	75.8 (74.0 to 77.5)	74.2 (72.5 to 75.7)	75.1 (73.4 to 76.8)	73.1 (71.1 to 75.0)
**Insurance**					
Have insurance	83.7 (83.0 to 84.3)	83.8 (83.4 to 84.2)	82.9 (82.6 to 83.3)	80.5 (79.9 to 81.1)	78.3 (77.7 to 78.9)
No insurance	58.1 (55.2 to 60.9)	55.4 (53.3 to 57.5)	49.6 (47.9 to 51.3)	54.7 (51.6 to 57.7)	38.2 (34.5 to 42.1)
**Primary HCP**					
Have an HCP	84.3 (83.7 to 84.9)	83.9 (83.6 to 84.3)	82.6 (82.3 to 83.0)	81.7 (81.2 to 82.3)	79.2 (78.6 to 79.8)
No HCP	51.4 (47.9 to 54.8)	50.9 (48.9 to 53.0)	45.2 (43.3 to 47.1)	48.3 (45.9 to 50.6)	43.2 (40.0 to 46.4)
**General health**					

© 2026 Al Hasan SM et al. *JAMA Network Open*.

Good health	82.6 (81.9 to 83.3)	82.8 (82.3 to 83.2)	81.6 (81.2 to 82.0)	80.7 (80.1 to 81.3)	78.8 (78.1 to 79.5)
Poor health	76.8 (75.4 to 78.2)	75.5 (74.4 to 76.5)	73.3 (72.4 to 74.2)	73.3 (71.8 to 74.7)	70.0 (68.6 to 71.4)
**Physical activity**					
Yes	83.7 (82.9 to 84.4)	83.5 (83.0 to 84.0)	82.2 (81.8 to 82.6)	81.1 (80.5 to 81.7)	79.1 (78.4 to 79.7)
No	75.6 (74.3 to 76.8)	75.5 (74.7 to 76.3)	73.5 (72.7 to 74.3)	73.5 (72.1 to 74.8)	71.1 (69.8 to 72.4)
**Smoking status**					
Current smoker	69.2 (67.2 to 71.1)	66.5 (65.3 to 67.8)	65.2 (64.1 to 66.3)	65.4 (63.7 to 67.0)	63.4 (61.7 to 65.1)
Former smoker	84.4 (83.3 to 85.4)	83.5 (82.8 to 84.1)	82.2 (81.5 to 82.8)	79.7 (78.7 to 80.6)	76.4 (75.2 to 77.6)
Never smoked	83.4 (82.5 to 84.2)	83.8 (83.2 to 84.3)	82.4 (81.9 to 82.9)	81.8 (81.0 to 82.6)	80.1 (79.3 to 80.9)
**Alcohol intake**					
Yes	85.0 (84.0 to 85.8)	84.7 (84.2 to 85.3)	83.4 (82.8 to 83.9)	81.6 (80.9 to 82.4)	80.0 (79.1 to 80.8)
No	78.7 (77.8 to 79.6)	78.5 (77.9 to 79.1)	76.9 (76.3 to 77.4)	76.6 (75.7 to 77.5)	74.4 (73.5 to 75.3)

[a]The prevalence of mammography use is reported as a weighted percentage, %, with 95% confidence interval (CI).

[b]Other races included Native Hawaiian/other Pacific Islander and races and ethnicities not specified and termed as “Others” in the BRFSS dataset.

[c]Employed for wages includes the employed for wages and the self-employed.

[d]Out of the work group included women who were out for work, both less than 1 year and more than 1 year.

[e]Other employment groups include students, the retired, and women unable to work.

[f]Separated includes divorced, widowed, and separated women.

[g]Not married includes never married and a member of an unmarried couple.

HCP stands for healthcare provider

© 2026 Al Hasan SM et al. *JAMA Network Open*.

eTable 5: Prevalence of mammography use among US women aged 40–49 years by states, 2002, 2008, 2010, 2018, and 2022

	40–49 years, % (95% CI)[a]				
**States**	**BRFSS 2002**	**BRFSS 2008**	**BRFSS 2010**	**BRFSS 2018**	**BRFSS 2022**
Alabama	78.1 (73.2 to 82.3)	67.0 (62.6 to 71.1)	68.6 (64.1 to 72.8)	64.0 (58.5 to 69.1)	61.6 (53.0 to 69.4)
Alaska	59.9 (52.2 to 67.1)	56.4 (48.3 to 64.2)	66.3 (56.6 to 74.7)	48.6 (39.2 to 58.1)	53.9 (47.2 to 60.4)
Arizona	70.6 (63.4 to 76.9)	68.1 (60.6 to 74.7)	67.9 (60.7 to 74.3)	62.6 (55.4 to 69.3)	54.7 (48.4 to 61.0)
Arkansas	67.9 (63.0 to 72.4)	62.6 (57.5 to 67.5)	58.2 (51.4 to 64.7)	54.0 (46.9 to 60.9)	59.7 (53.1 to 66.0)
California	66.3 (60.8 to 71.4)	70.3 (67.0 to 73.4)	71.8 (69.2 to 74.2)	57.4 (52.8 to 61.9)	52.4 (46.7 to 58.1)
Colorado	67.0 (62.1 to 71.5)	66.1 (63.1 to 68.9)	62.3 (59.0 to 65.5)	51.0 (46.1 to 55.9)	55.2 (50.6 to 59.7)
Connecticut	78.2 (74.1 to 81.8)	81.6 (77.7 to 85.0)	75.7 (71.3 to 79.6)	72.0 (67.4 to 76.1)	70.9 (65.6 to 75.7)
Delaware	78.4 (72.4 to 83.3)	78.2 (73.1 to 82.6)	76.7 (71.6 to 81.2)	69.3 (62.3 to 75.5)	61.4 (52.4 to 69.6)
District of Columbia	76.1 (68.2 to 82.6)	71.3 (65.9 to 76.1)	71.5 (65.5 to 76.7)	61.1 (54.0 to 67.8)	57.7 (49.9 to 65.1)
Florida	72.7 (68.8 to 76.3)	71.5 (67.0 to 75.5)	69.3 (65.7 to 72.6)	68.6 (62.3 to 74.2)	60.0 (52.5 to 67.1)
Georgia	69.1 (64.5 to 73.3)	69.8 (65.1 to 74.2)	71.2 (66.3 to 75.7)	60.4 (55.6 to 65.0)	60.6 (54.5 to 66.3)
Hawaii	63.7 (59.3 to 67.9)	70.7 (65.9 to 75.1)	71.7 (66.6 to 76.2)	72.7 (67.4 to 77.4)	65.4 (59.9 to 70.4)
Idaho	59.3 (54.6 to 63.8)	57.8 (52.8 to 62.6)	53.4 (48.1 to 58.6)	46.3 (37.7 to 55.1)	48.7 (43.5 to 54.0)
Illinois	69.5 (62.7 to 75.5)	72.2 (68.1 to 75.9)	68.3 (63.0 to 73.1)	66.4 (60.6 to 71.7)	55.5 (48.8 to 62.0)
Indiana	67.1 (63.1 to 70.8)	70.2 (65.0 to 74.8)	63.3 (59.4 to 67.0)	61.2 (55.8 to 66.3)	58.1 (53.8 to 62.2)
Iowa	70.2 (64.5 to 75.4)	69.1 (64.8 to 73.1)	72.4 (67.9 to 76.6)	65.4 (61.2 to 69.5)	58.3 (53.2 to 63.3)
Kansas	66.7 (62.4 to 70.7)	66.5 (63.0 to 69.8)	67.2 (63.4 to 70.8)	57.3 (52.7 to 61.7)	57.1 (52.3 to 61.8)
Kentucky	72.3 (67.5 to 76.7)	69.0 (65.1 to 72.6)	60.1 (54.5 to 65.5)	52.6 (46.2 to 59.0)	57.9 (49.8 to 65.5)
Louisiana	69.6 (64.8 to 74.1)	68.1 (64.0 to 71.9)	71.1 (67.3 to 74.6)	64.3 (57.6 to 70.5)	67.8 (61.4 to 73.5)
Maine	76.7 (71.3 to 81.3)	78.6 (74.8 to 81.9)	72.5 (68.7 to 76.0)	61.7 (55.7 to 67.3)	59.6 (54.4 to 64.5)
Maryland	79.2 (74.8 to 83.0)	69.2 (65.6 to 72.6)	73.4 (69.8 to 76.8)	70.2 (66.4 to 73.7)	66.4 (62.1 to 70.4)
Massachusetts	78.3 (74.9 to 81.3)	79.5 (77.0 to 81.7)	76.5 (73.3 to 79.4)	68.2 (61.9 to 73.9)	64.3 (59.8 to 68.5)
Michigan	72.1 (67.8 to 75.9)	71.9 (68.6 to 75.0)	71.1 (67.2 to 74.7)	62.5 (57.7 to 67.1)	66.2 (61.2 to 70.9)
Minnesota	74.8 (70.5 to 78.7)	77.4 (73.1 to 81.1)	75.1 (71.1 to 78.7)	64.3 (61.0 to 67.5)	62.0 (58.4 to 65.5)
Mississippi	60.6 (55.7 to 65.3)	63.0 (58.7 to 67.1)	61.3 (56.9 to 65.5)	60.4 (54.8 to 65.7)	65.4 (58.6 to 71.7)
Missouri	70.7 (65.4 to 75.4)	64.1 (58.9 to 69.0)	64.2 (58.1 to 69.8)	60.8 (53.9 to 67.3)	63.6 (57.4 to 69.3)
Montana	65.5 (59.6 to 70.9)	64.7 (60.1 to 69.1)	58.3 (53.2 to 63.2)	49.4 (42.0 to 56.7)	53.7 (48.1 to 59.3)
Nebraska	72.5 (67.8 to 76.7)	67.2 (63.4 to 70.8)	68.9 (64.8 to 72.7)	62.0 (57.4 to 66.4)	55.2 (49.3 to 60.9)
Nevada	65.1 (57.6 to 71.9)	59.9 (53.2 to 66.3)	61.8 (54.3 to 68.7)	59.4 (49.3 to 68.8)	49.1 (38.3 to 60.0)
New Hampshire	77.7 (73.9 to 81.0)	78.4 (75.0 to 81.4)	74.5 (70.2 to 78.3)	63.7 (57.2 to 69.7)	65.3 (57.7 to 72.1)
New Jersey	75.5 (69.4 to 80.7)	72.0 (68.7 to 75.1)	74.4 (71.3 to 77.2)	62.8 (50.1 to 73.9)	66.2 (60.4 to 71.5)
New Mexico	59.5 (54.8 to 64.0)	64.2 (59.5 to 68.6)	59.2 (54.2 to 64.1)	51.6 (45.5 to 57.7)	44.7 (37.6 to 51.9)
New York	73.5 (68.9 to 77.6)	74.4 (70.8 to 77.7)	71.5 (67.8 to 74.9)	67.6 (64.1 to 70.9)	66.4 (62.2 to 70.3)

© 2026 Al Hasan SM et al. *JAMA Network Open*.

North Carolina	77.0 (72.5 to 81.0)	70.3 (67.3 to 73.1)	68.3 (64.7 to 71.6)	64.7 (58.0 to 70.9)	62.8 (55.9 to 69.2)
North Dakota	71.1 (65.8 to 75.8)	64.6 (59.8 to 69.2)	67.2 (62.1 to 71.9)	56.9 (49.0 to 64.5)	63.3 (55.8 to 70.2)
Ohio	69.8 (64.7 to 74.3)	68.8 (65.4 to 72.0)	66.4 (62.5 to 70.0)	69.9 (65.4 to 74.1)	57.2 (53.0 to 61.3)
Oklahoma	65.4 (61.5 to 69.1)	61.2 (57.4 to 64.9)	61.7 (57.4 to 65.8)	56.9 (50.5 to 63.0)	53.5 (47.9 to 58.9)
Oregon	64.8 (59.1 to 70.0)	65.8 (60.9 to 70.3)	59.5 (54.1 to 64.7)	59.1 (53.4 to 64.6)	54.6 (49.2 to 59.9)
Pennsylvania	72.1 (69.1 to 74.8)	69.8 (66.1 to 73.2)	67.0 (63.2 to 70.5)	64.6 (58.6 to 70.1)	66.3 (58.4 to 73.4)
Rhode Island	79.8 (75.6 to 83.4)	73.2 (68.8 to 77.3)	76.0 (72.0 to 79.6)	66.9 (60.0 to 73.1)	68.7 (61.9 to 74.8)
South Carolina	67.7 (62.3 to 72.6)	64.3 (59.4 to 68.8)	63.1 (58.1 to 67.8)	54.7 (49.7 to 59.7)	60.5 (55.2 to 65.6)
South Dakota	67.6 (63.1 to 71.9)	64.4 (59.8 to 68.8)	71 (66.1 to 75.4)	69.1 (59.6 to 77.2)	77.7 (67.7 to 85.3)
Tennessee	72.2 (66.5 to 77.4)	66.4 (60.6 to 71.7)	69.5 (64.2 to 74.3)	60.2 (52.9 to 67.0)	60.8 (54.0 to 67.2)
Texas	59.4 (55.3 to 63.3)	67.0 (63.1 to 70.6)	65.3 (61.4 to 69)	60.5 (53.2 to 67.4)	54.4 (48.5 to 60.2)
Utah	58.8 (52.3 to 65.1)	58.1 (52.8 to 63.3)	55.7 (51.9 to 59.5)	51.2 (47.0 to 55.4)	53.0 (48.7 to 57.3)
Vermont	69.3 (65.1 to 73.3)	76.0 (72.6 to 79.1)	69.6 (65.7 to 73.2)	53.6 (47.4 to 59.7)	49.3 (44.3 to 54.2)
Virginia	70.2 (64.2 to 75.5)	71.6 (66.2 to 76.5)	74.7 (69.3 to 79.5)	67.4 (62.7 to 71.9)	62.7 (57.7 to 67.4)
Washington	65.7 (61.0 to 70.1)	66.3 (63.9 to 68.6)	67.3 (64.5 to 69.9)	47.9 (43.4 to 52.5)	50.6 (47.8 to 53.5)
West Virginia	71.6 (66.2 to 76.4)	64.3 (59.0 to 69.3)	64.5 (59 to 69.5)	64.8 (58.8 to 70.5)	60.4 (54.4 to 66.1)
Wisconsin	75.9 (71.4 to 79.8)	71.5 (66.8 to 75.8)	75.2 (70.1 to 79.6)	65.8 (57.7 to 73.0)	58.3 (53.5 to 62.9)
Wyoming	60.8 (55.6 to 65.7)	57.9 (54.1 to 61.6)	58.8 (53.7 to 63.7)	47.7 (40.7 to 54.9)	50.6 (43.5 to 57.8)

[a]The prevalence of mammography use is reported as a weighted percentage, %, with 95% confidence interval (CI).

© 2026 Al Hasan SM et al. *JAMA Network Open*.

eTable 6: Prevalence of mammography use among US women aged 50–74 years by states, 2002, 2008, 2010, 2018, and 2022

	50–74 years, % (95% CI)[a]				
**States**	**BRFSS 2002**	**BRFSS 2008**	**BRFSS 2010**	**BRFSS 2018**	**BRFSS 2022**
Alabama	81.2 (77.9 to 84.2)	77.3 (74.9 to 79.6)	77.7 (75.4 to 79.8)	80.4 (77.9 to 82.7)	76.4 (73.0 to 79.4)
Alaska	82.2 (75.6 to 87.3)	74.9 (69.6 to 79.6)	74.5 (68.7 to 79.5)	67.0 (61.5 to 72.1)	68.7 (64.9 to 72.4)
Arizona	82.6 (78.8 to 85.8)	82.1 (78.5 to 85.2)	78.1 (74.3 to 81.5)	73.1 (69.7 to 76.3)	74.9 (72.1 to 77.4)
Arkansas	70.4 (66.9 to 73.6)	76.5 (74.1 to 78.8)	75.1 (72.1 to 77.9)	72.7 (69.7 to 75.5)	75.0 (72.0 to 77.7)
California	81.4 (77.1 to 85.0)	83.7 (81.9 to 85.5)	83.3 (81.9 to 84.7)	81.1 (78.6 to 83.3)	76.5 (73.4 to 79.4)
Colorado	81.1 (78.0 to 83.9)	77.0 (75.3 to 78.6)	75.9 (74.0 to 77.6)	71.2 (68.9 to 73.5)	71.1 (68.2 to 74.0)
Connecticut	86.5 (83.6 to 88.9)	88.0 (85.8 to 89.9)	86.0 (83.9 to 87.9)	83.0 (81.0 to 84.9)	81.7 (79.0 to 84.0)
Delaware	88.8 (86.1 to 91.0)	87.4 (85.0 to 89.5)	84.4 (81.8 to 86.6)	84.2 (81.5 to 86.5)	79.7 (76.4 to 82.6)
District of Columbia	86.1 (81.7 to 89.6)	85.4 (82.8 to 87.6)	85.8 (82.9 to 88.2)	79.9 (76.8 to 82.6)	77.4 (72.7 to 81.6)
Florida	81.1 (78.3 to 83.5)	82.7 (80.4 to 84.8)	80.8 (79.0 to 82.4)	81.4 (79.0 to 83.5)	78.0 (74.9 to 80.8)
Georgia	80.6 (77.3 to 83.4)	85.3 (83.3 to 87.2)	81.8 (79.4 to 83.9)	80.0 (77.9 to 81.9)	76.3 (73.5 to 78.9)
Hawaii	76.1 (72.6 to 79.3)	83.4 (81.2 to 85.4)	80.9 (78.4 to 83.1)	87.0 (85.0 to 88.8)	78.5 (75.6 to 81.2)
Idaho	73.0 (69.8 to 76.0)	72.8 (70.0 to 75.5)	69.5 (67.0 to 71.8)	68.2 (63.9 to 72.2)	68.5 (65.5 to 71.4)
Illinois	77.3 (72.7 to 81.4)	80.0 (77.5 to 82.3)	76.4 (73.6 to 78.9)	78.7 (75.5 to 81.6)	72.8 (68.4 to 76.8)
Indiana	79.6 (76.9 to 82.0)	76.7 (73.7 to 79.5)	77.3 (75.3 to 79.1)	76.6 (74.3 to 78.8)	78.1 (76.1 to 80.1)
Iowa	83.1 (80.0 to 85.8)	80.8 (78.6 to 82.9)	80.4 (78.2 to 82.4)	80.8 (78.9 to 82.5)	79.7 (77.2 to 81.9)
Kansas	82.6 (79.9 to 85.1)	79.9 (78.1 to 81.6)	80.9 (79.1 to 82.5)	74.6 (72.6 to 76.5)	74.5 (72.1 to 76.7)
Kentucky	80.2 (77.7 to 82.6)	79.3 (77.4 to 81.1)	76.0 (73.8 to 78.2)	77.6 (74.7 to 80.3)	72.9 (68.6 to 76.8)
Louisiana	80.9 (78.2 to 83.3)	80.4 (78.3 to 82.3)	79.2 (77.3 to 81.0)	82.7 (80.0 to 85.1)	82.5 (79.9 to 84.9)
Maine	87.0 (83.6 to 89.7)	86.6 (84.9 to 88.2)	84.9 (83.3 to 86.4)	81.0 (79.0 to 83.0)	81.6 (79.6 to 83.5)
Maryland	85.9 (82.7 to 88.7)	81.5 (79.4 to 83.5)	85.6 (83.8 to 87.3)	81.2 (79.5 to 82.8)	83.2 (81.4 to 84.8)
Massachusetts	88.6 (86.4 to 90.5)	89.8 (88.6 to 90.8)	89.4 (88.0 to 90.6)	86.7 (84.4 to 88.7)	84.9 (82.8 to 86.9)
Michigan	83.3 (79.9 to 86.1)	84.0 (82.4 to 85.5)	82.1 (80.3 to 83.8)	80.0 (77.9 to 82.0)	77.7 (75.5 to 79.7)
Minnesota	86.5 (83.9 to 88.8)	81.5 (78.9 to 83.9)	85.6 (83.7 to 87.4)	82.3 (80.8 to 83.7)	79.6 (77.7 to 81.3)
Mississippi	73.8 (70.7 to 76.7)	72.2 (70.1 to 74.2)	72.3 (70.1 to 74.3)	70.5 (67.6 to 73.3)	73.1 (69.1 to 76.8)
Missouri	77.2 (73.7 to 80.4)	78.8 (75.9 to 81.4)	75.4 (72.2 to 78.2)	75.2 (72.2 to 78.0)	75.0 (72.0 to 77.8)
Montana	77.8 (74.2 to 81.0)	75.5 (73.1 to 77.7)	72.7 (70.2 to 75.0)	74.1 (70.7 to 77.2)	74.8 (72.0 to 77.5)
Nebraska	78.5 (75.4 to 81.3)	77.8 (75.8 to 79.8)	75.9 (73.8 to 77.9)	75.4 (73.3 to 77.3)	76.8 (74.1 to 79.3)
Nevada	78.8 (73.4 to 83.3)	72.3 (68.6 to 75.7)	71.7 (67.6 to 75.6)	72.4 (67.6 to 76.7)	70.5 (65.1 to 75.4)
New Hampshire	84.9 (82.3 to 87.3)	86.9 (85.1 to 88.6)	84.7 (82.7 to 86.5)	83.0 (80.6 to 85.1)	80.8 (78.4 to 83.0)
New Jersey	80.7 (76.1 to 84.5)	79.7 (77.9 to 81.5)	81.1 (79.3 to 82.9)	81.3 (76.1 to 85.6)	76.3 (73.0 to 79.2)
New Mexico	76.8 (73.7 to 79.6)	74.7 (72.1 to 77.1)	76.4 (74.2 to 78.5)	71.7 (68.8 to 74.4)	69.4 (65.6 to 73.0)
New York	85.5 (82.5 to 88.1)	84.0 (81.9 to 85.9)	81.9 (80.0 to 83.5)	82.1 (80.2 to 83.8)	79.2 (77.0 to 81.3)

© 2026 Al Hasan SM et al. *JAMA Network Open*.

North Carolina	85.1 (82.2 to 87.6)	83.2 (81.8 to 84.5)	82.1 (80.3 to 83.7)	79.8 (76.4 to 82.8)	79.1 (74.9 to 82.7)
North Dakota	81.2 (77.6 to 84.3)	82.0 (79.6 to 84.1)	79.4 (76.9 to 81.6)	79.2 (76.4 to 81.8)	80.2 (77.1 to 83.1)
Ohio	81.2 (77.9 to 84.1)	80.2 (78.5 to 81.7)	78.2 (76.2 to 80.0)	77.6 (75.6 to 79.6)	75.6 (73.5 to 77.6)
Oklahoma	73.6 (71.1 to 75.9)	73.2 (71.1 to 75.2)	70.7 (68.6 to 72.7)	74.3 (71.4 to 77.1)	69.3 (66.2 to 72.3)
Oregon	81.4 (77.9 to 84.4)	82.1 (79.8 to 84.2)	76.9 (74.4 to 79.1)	78.3 (75.5 to 80.8)	78.0 (74.9 to 80.9)
Pennsylvania	81.4 (79.4 to 83.2)	81.5 (79.6 to 83.1)	78.8 (76.9 to 80.5)	78.7 (75.7 to 81.4)	75.7 (71.1 to 79.7)
Rhode Island	90.7 (88.2 to 92.7)	87.1 (85.0 to 88.9)	86.3 (84.4 to 88.0)	86.9 (84.7 to 88.9)	86.0 (83.6 to 88.1)
South Carolina	80.1 (76.9 to 83.0)	80.0 (77.7 to 82.1)	80.7 (78.5 to 82.7)	76.9 (74.9 to 78.9)	79.7 (77.6 to 81.6)
South Dakota	81.2 (78.5 to 83.7)	80.9 (78.6 to 83.0)	81.0 (78.8 to 83.0)	82.0 (78.7 to 84.9)	72.8 (64.6 to 79.7)
Tennessee	79.8 (76.2 to 82.9)	79.2 (76.6 to 81.5)	79.8 (77.3 to 82.1)	76.4 (73.0 to 79.5)	75.1 (71.6 to 78.3)
Texas	76.7 (73.7 to 79.6)	76.7 (74.4 to 78.9)	73.7 (71.6 to 75.6)	74.9 (70.0 to 79.3)	73.8 (70.6 to 76.7)
Utah	79.4 (75.3 to 82.9)	73.3 (69.9 to 76.4)	73.5 (71.3 to 75.6)	72.3 (69.7 to 74.7)	74.6 (71.9 to 77.1)
Vermont	84.1 (81.3 to 86.6)	82.7 (80.8 to 84.5)	82.9 (81.0 to 84.6)	77.0 (74.4 to 79.5)	75.6 (73.1 to 78.0)
Virginia	79.4 (75.4 to 82.9)	82.8 (80.2 to 85.1)	79.8 (76.8 to 82.5)	81.1 (79.0 to 83.0)	77.4 (74.9 to 79.7)
Washington	81.6 (78.5 to 84.3)	81.3 (80.2 to 82.5)	79.1 (77.8 to 80.4)	75.1 (73.1 to 77.1)	75.4 (73.9 to 76.8)
West Virginia	77.9 (74.6 to 80.9)	79.6 (77.1 to 81.9)	76.9 (74.4 to 79.3)	75.0 (72.1 to 77.7)	76.2 (73.3 to 78.8)
Wisconsin	85.1 (82.3 to 87.6)	79.9 (77.1 to 82.4)	81.5 (78.7 to 84.1)	78.2 (75.0 to 81.1)	82.1 (80.1 to 83.9)
Wyoming	73.6 (70.1 to 76.8)	72.8 (70.7 to 74.8)	71.9 (69.5 to 74.2)	68.1 (65.0 to 71.1)	65.2 (61.7 to 68.4)

[a]The prevalence of mammography use is reported as a weighted percentage, %, with 95% confidence interval.

© 2026 Al Hasan SM et al. *JAMA Network Open*.

eTable 7: Biennial changes in mammography use by states before and after the 2009 USPSTF breast cancer screening recommendation

	**40–49 years, ABPC (95% CI)[a]**		**50–74 years, ABPC (95% CI)[a]**	
	**Before the 2009 policy**	**Following the 2009 policy**	**Before the 2009 policy**	**Following the 2009 policy**
**States**	**2002–2008**	**2010–2022**	**2002–2008**	**2010–2022**
Alabama	–1.95 (–5.10 to 1.31)	–0.14 (–1.31 to 1.04)	–0.62 (–1.16 to –0.08)	–0.18 (–0.80 to 0.45)
Alaska	0.96 (–1.73 to 3.72)	–1.64 (–3.51 to 0.27)	–1.40 (–1.98 to –0.82)	–0.64 (–1.60 to 0.33)
Arizona	0.06 (–1.19 to 1.32)	–0.52 (–3.84 to 2.90)	–0.50 (–1.34 to 0.34)	–0.17 (–0.54 to 0.21)
Arkansas	–1.72 (–4.78 to 1.44)	0.16 (–0.70 to 1.03)	1.29 (–3.04 to 5.82)	0.80 (0.07 to 1.53)
California	0.89 (–0.35 to 2.16)	–2.47 (–4.33 to –0.57)	0.15 (–0.20 to 0.50)	–0.65 (–1.20 to –0.09)
Colorado	0.09 (–3.29 to 3.58)	–1.12 (–2.04 to –0.18)	–0.71 (–1.17 to –0.25)	–0.40 (–0.73 to –0.08)
Connecticut	0.36 (0.04 to 0.68)	–0.73 (–0.85 to –0.61)	0.22 (–0.16 to 0.60)	–0.29 (–0.91 to 0.32)
Delaware	–0.26 (–0.92 to 0.40)	–1.45 (–2.25 to –0.65)	–0.39 (–0.76 to –0.03)	–0.71 (–1.95 to 0.55)
District of Columbia	–0.82 (–1.61 to –0.02)	–1.63 (–2.47 to –0.79)	0.01 (–0.90 to 0.92)	–0.75 (–1.32 to –0.18)
Florida	0.09 (–0.85 to 1.04)	–0.78 (–5.24 to 3.90)	0.17 (–0.28 to 0.63)	–0.14 (–1.85 to 1.60)
Georgia	0.34 (–0.62 to 1.31)	–0.96 (–2.31 to 0.40)	0.90 (–0.20 to 2.02)	–0.62 (–0.92 to –0.33)
Hawaii	2.00 (–0.37 to 4.43)	–1.04 (–1.73 to –0.34)	0.97 (0.54 to 1.41)	–0.08 (–1.11 to 0.96)
Idaho	–0.97 (–2.06 to 0.13)	–0.32 (–2.63 to 2.04)	–0.37 (–1.03 to 0.30)	0.10 (–1.10 to 1.31)
Illinois	0.02 (–0.62 to 0.66)	–1.60 (–4.17 to 1.05)	–0.60 (–1.81 to 0.62)	–0.20 (–1.08 to 0.70)
Indiana	0.42 (–3.13 to 4.10)	–0.78 (–1.93 to 0.39)	–0.68 (–4.42 to 3.22)	0.82 (0.08 to 1.56)
Iowa	0.37 (–1.39 to 2.16)	–1.28 (–2.26 to –0.29)	–0.31 (–0.53 to –0.10)	0.00 (–0.30 to 0.31)
Kansas	–0.21 (–0.78 to 0.36)	–1.13 (–1.63 to –0.64)	–0.25 (–1.01 to 0.53)	–0.68 (–1.09 to –0.27)
Kentucky	–1.57 (–3.80 to 0.72)	–0.64 (–2.71 to 1.48)	–0.31 (–0.76 to 0.15)	–0.57 (–2.48 to 1.39)
Louisiana	0.37 (–1.46 to 2.23)	0.00 (–1.24 to 1.25)	–0.08 (–0.83 to 0.68)	0.58 (0.15 to 1.02)
Maine	0.19 (–1.36 to 1.76)	–1.86 (–2.32 to –1.39)	–0.34 (–0.59 to –0.09)	–0.16 (–0.7 to 0.39)
Maryland	–1.50 (–5.38 to 2.54)	–0.79 (–1.58 to 0.00)	–0.33 (–0.73 to 0.08)	–0.07 (–0.92 to 0.78)
Massachusetts	0.39 (–1.19 to 2.00)	–1.99 (–2.61 to –1.37)	0.35 (–0.38 to 1.09)	–0.49 (–0.68 to –0.31)
Michigan	–0.58 (–1.03 to –0.13)	–0.19 (–1.71 to 1.36)	0.11 (–1.17 to 1.40)	–0.45 (–0.64 to –0.27)
Minnesota	0.86 (–2.17 to 3.99)	–1.50 (–2.30 to –0.71)	–0.31 (–0.63 to 0.01)	–0.48 (–1.27 to 0.32)
Mississippi	0.42 (–0.89 to 1.74)	1.27 (0.36 to 2.19)	–0.27 (–0.63 to 0.09)	0.12 (–0.92 to 1.17)
Missouri	–1.25 (–4.72 to 2.35)	0.34 (–0.46 to 1.14)	0.18 (–2.19 to 2.61)	–0.15 (–0.7 to 0.41)
Montana	–1.17 (–2.37 to 0.04)	–0.57 (–3.97 to 2.94)	–0.96 (–1.66 to –0.25)	0.46 (0.09 to 0.84)
Nebraska	–1.23 (–4.12 to 1.75)	–0.40 (–1.18 to 0.39)	–0.68 (–1.07 to –0.28)	0.03 (–0.69 to 0.75)
Nevada	0.03 (–1.57 to 1.66)	–0.90 (–6.95 to 5.54)	–1.16 (–2.02 to –0.28)	–0.25 (–1.20 to 0.71)
New Hampshire	0.02 (–1.15 to 1.19)	–1.64 (–3.85 to 0.63)	0.22 (–1.10 to 1.55)	–0.62 (–0.95 to –0.28)
New Jersey	0.57 (–1.93 to 3.13)	–1.91 (–3.49 to –0.30)	0.04 (–0.33 to 0.40)	–0.26 (–1.30 to 0.78)
New Mexico	1.14 (–1.21 to 3.54)	–2.74 (–3.70 to –1.77)	–0.27 (–2.50 to 2.00)	0.00 (–0.50 to 0.50)

© 2026 Al Hasan SM et al. *JAMA Network Open*.

New York	0.57 (–2.67 to 3.92)	–1.23 (–2.09 to –0.36)	–0.32 (–1.06 to 0.43)	0.04 (–0.60 to 0.68)
North Carolina	–1.04 (–2.34 to 0.28)	–0.17 (–1.37 to 1.04)	–0.05 (–0.99 to 0.91)	–0.26 (–0.46 to –0.05)
North Dakota	–0.61 (–1.60 to 0.38)	–0.04 (–3.27 to 3.29)	–0.21 (–0.79 to 0.37)	0.30 (–0.56 to 1.17)
Ohio	0.22 (–0.54 to 0.98)	–1.40 (–3.28 to 0.52)	–0.52 (–1.04 to 0.01)	–0.18 (–0.64 to 0.29)
Oklahoma	–1.05 (–3.29 to 1.24)	–0.05 (–0.68 to 0.58)	–0.56 (–1.92 to 0.81)	0.15 (–0.77 to 1.07)
Oregon	–0.06 (–3.10 to 3.08)	–1.04 (–2.05 to –0.02)	–0.15 (–0.94 to 0.64)	0.32 (–0.91 to 1.57)
Pennsylvania	–1.03 (–2.84 to 0.81)	0.18 (–0.40 to 0.76)	–0.36 (–0.78 to 0.06)	–0.05 (–1.26 to 1.17)
Rhode Island	–0.62 (–1.24 to 0.01)	–1.11 (–2.72 to 0.52)	–0.58 (–1.24 to 0.08)	0.06 (–0.34 to 0.46)
South Carolina	–0.86 (–1.39 to –0.33)	–0.15 (–2.08 to 1.82)	0.23 (–0.16 to 0.63)	0.45 (–0.72 to 1.62)
South Dakota	–0.47 (–4.14 to 3.34)	2.17 (0.63 to 3.73)	0.12 (–0.04 to 0.28)	–0.36 (–1.23 to 0.50)
Tennessee	–1.40 (–2.40 to –0.39)	–0.69 (–2.87 to 1.53)	0.18 (–1.10 to 1.49)	–0.37 (–0.62 to –0.12)
Texas	2.42 (–1.24 to 6.21)	–1.18 (–2.26 to –0.09)	–0.23 (–1.56 to 1.12)	0.10 (–0.72 to 0.94)
Utah	–1.70 (–3.18 to –0.19)	–0.96 (–4.36 to 2.56)	–0.99 (–4.64 to 2.80)	–0.02 (–0.59 to 0.56)
Vermont	1.60 (–0.12 to 3.36)	–3.15 (–3.86 to –2.43)	0.06 (–0.83 to 0.95)	–0.82 (–1.36 to –0.28)
Virginia	1.18 (–1.30 to 3.73)	–0.89 (–2.05 to 0.29)	0.11 (–0.19 to 0.41)	–0.29 (–0.94 to 0.37)
Washington	0.82 (–0.56 to 2.21)	–1.60 (–2.89 to –0.30)	–0.07 (–1.09 to 0.96)	–0.28 (–0.51 to –0.05)
West Virginia	–1.55 (–4.97 to 1.98)	0.30 (–0.51 to 1.12)	0.25 (–1.85 to 2.40)	–0.20 (–0.56 to 0.16)
Wisconsin	0.14 (–1.04 to 1.32)	–1.24 (–3.68 to 1.26)	–0.69 (–1.09 to –0.29)	–0.14 (–1.09 to 0.81)
Wyoming	–0.46 (–4.52 to 3.78)	0.45 (–1.18 to 2.10)	–0.36 (–0.91 to 0.19)	–0.44 (–2.08 to 1.23)

[a]The average biennial percentage change (ABPC) was calculated as a geometric weighted average of the biennial percent changes of various segments of mammography use prevalence trends in the US from 2002–2022. The estimates were adjusted for the methodological changes in the BFRSS survey methods in 2011.

© 2026 Al Hasan SM et al. *JAMA Network Open*.

eTable 8: Differences in biennial changes in mammography use between 2012–2022 and 2012–2018

	40–49 years, ABPC (95% CI)[a]			50–74 years, ABPC (95% CI)[a]		
**Characteristics**	**2012–2022**	**2012–2018**	**Differences[b]**	**2012–2022**	**2012–2018**	**Differences[b]**
**Overall**	–1.22 (–1.87 to –0.73)	–1.07 (–2.14 to –0.21)	–0.15 (–1.27 to 0.97)	–0.14 (–0.36 to 0.08)	0.02 (–0.23 to 0.25)	–0.16 (–0.48 to 0.17)
**Race and ethnicity**						
American Indian/AN	0.03 (–3.77 to 3.21)	0.02 (–6.70 to 7.24)	0.01 (–7.78 to 7.80)	–1.15 (–1.30 to –1.06)	1.12 (1.02 to 1.23)	–2.27 (–2.43 to –2.11)
Asian	–3.10 (–7.65 to 0.47)	–4.45 (–8.31 to –1.14)	1.35 (–4.07 to 6.77)	–1.01 (–1.97 to 0.02)	–0.99 (–1.56 to –0.54)	–0.02 (–1.14 to 1.10)
Hispanic or Latino	–1.47 (–4.69 to 0.94)	–0.02 (–2.92 to 2.68)	–1.45 (–5.42 to 2.52)	–0.27 (–0.79 to 0.22)	0.66 (–0.73 to 1.98)	–0.93 (–2.37 to 0.52)
Non-Hispanic Black	–0.54 (–1.15 to 0.02)	–0.27 (–1.03 to 0.41)	–0.27 (–1.20 to 0.66)	0.11 (–0.21 to 0.39)	0.24 (0.00 to 0.47)	–0.13 (–0.51 to 0.25)
Non-Hispanic White	–1.00 (–1.28 to –0.78)	–1.25 (–1.67 to –0.91)	0.24 (–0.21 to 0.70)	–0.13 (–0.39 to 0.10)	–0.11 (–0.62 to 0.37)	–0.02 (–0.57 to 0.54)
Other[c] or multiracial	–1.61 (–3.32 to 0.14)	–1.78 (–2.69 to 0.97)	0.17 (–2.34 to 2.69)	–0.58 (–1.73 to 0.35)	1.45 (0.00 to 3.15)	–2.04 (–3.92 to –0.15)
**Education**						
Below high school	–1.53 (–7.94 to 3.33)	0.93 (–2.90 to 4.81)	–2.46 (–9.29 to 4.37)	–0.36 (–1.37 to 0.44)	0.97 (–0.39 to 2.16)	–1.33 (–2.89 to 0.24)
High school graduate	–1.62 (–3.38 to –0.49)	–1.54 (–3.95 to 0.23)	–0.08 (–2.63 to 2.46)	–0.26 (–0.56 to 0.04)	–0.05 (–0.35 to 0.22)	–0.21 (–0.62 to 0.21)
Some college	–1.49 (–1.63 to –1.38)	–1.18 (–1.41 to –0.99)	–0.31 (–0.55 to –0.06)	–0.23 (–0.37 to –0.12)	–0.18 (–0.45 to 0.07)	–0.06 (–0.34 to 0.23)
College graduate	–1.03 (–1.21 to –0.91)	–1.73 (–2.05 to –1.47)	0.70 (0.37 to 1.03)	–0.26 (–0.51 to –0.06)	–0.23 (–0.74 to 0.22)	–0.02 (–0.56 to 0.51)
**Employment**						
Employed for wages[d]	–1.36 (–1.92 to –0.95)	–1.31 (–2.65 to –0.25)	–0.05 (–1.35 to 1.24)	–0.27 (–0.45 to –0.14)	–0.13 (–0.48 to 0.17)	–0.14 (–0.49 to 0.22)
Out of work[e]	0.04 (–2.49 to 2.21)	–0.65 (–4.92 to 2.82)	0.69 (–3.84 to 5.22)	0.18 (–0.65 to 0.85)	0.62 (–0.36 to 1.44)	–0.44 (–1.61 to 0.73)
Homemaker	–1.36 (–2.48 to –0.48)	–1.01 (–1.89 to –0.22)	–0.35 (–1.66 to 0.95)	–0.45 (–1.38 to 0.20)	–0.14 (–1.27 to 0.71)	–0.31 (–1.58 to 0.95)
Others[f]	–1.43 (–2.27 to –0.75)	–0.33 (–1.86 to 1.10)	–1.09 (–2.75 to 0.57)	–0.13 (–0.56 to 0.24)	–0.06 (–0.47 to 0.33)	–0.07 (–0.63 to 0.50)
**Income**						
Less than $25,000	–1.22 (–4.39 to 1.21)	0.46 (–1.62 to 2.54)	–1.67 (–5.16 to 1.81)	–0.20 (–0.59 to 0.17)	0.77 (0.45 to 1.11)	–0.96 (–1.47 to –0.46)
$25,000 to $49,999	–1.83 (–3.35 to –0.64)	–1.84 (–4.42 to 0.57)	0.00 (–2.84 to 2.84)	–0.42 (–0.89 to –0.05)	–0.32 (–0.85 to 0.18)	–0.10 (–0.76 to 0.56)
More than $50,000	–1.50 (–1.72 to –1.34)	–1.78 (–2.15 to –1.48)	0.28 (–0.11 to 0.66)	–0.38 (–0.48 to –0.30)	–0.37 (–0.59 to –0.19)	–0.01 (–0.22 to 0.21)
**Marital status**						
Married	–1.24 (–2.05 to –0.65)	–1.14 (–2.43 to –0.07)	–0.10 (–1.47 to 1.27)	–0.15 (–0.33 to –0.01)	–0.19 (–0.43 to 0.01)	0.04 (–0.23 to 0.31)
Separated[g]	–0.78 (–1.27 to –0.37)	–0.36 (–1.13 to 0.30)	–0.42 (–1.27 to 0.42)	–0.20 (–0.82 to 0.43)	0.34 (–0.08 to 0.72)	–0.54 (–1.28 to 0.20)
Not married[h]	–1.54 (–2.25 to –0.93)	–1.75 (–3.17 to –0.48)	0.21 (–1.29 to 1.71)	–0.10 (–0.47 to 0.19)	0.21 (–0.68 to 1.10)	–0.31 (–1.25 to 0.64)
**Insurance**						
Have insurance	–1.50 (–1.82 to –1.24)	–1.78 (–2.28 to –1.38)	0.29 (–0.25 to 0.82)	–0.41 (–0.84 to –0.09)	–0.45 (–1.24 to 0.17)	0.04 (–0.76 to 0.83)
No insurance	–2.55 (–4.48 to –1.35)	1.52 (–0.17 to 2.95)	–4.08 (–6.29 to –1.86)	–0.21 (–3.66 to 3.35)	1.84 (0.65 to 2.81)	–2.05 (–5.72 to 1.61)
**Primary HCP**						
Have an HCP	–1.11 (–1.56 to –0.76)	–1.06 (–1.91 to –0.35)	–0.05 (–0.93 to 0.83)	–0.22 (–0.59 to 0.08)	–0.08 (–0.50 to 0.30)	–0.14 (–0.66 to 0.38)
No HCP	–2.37 (–6.89 to 1.28)	–0.20 (–2.17 to 1.60)	–2.17 (–6.67 to 2.33)	–0.21 (–0.96 to 0.36)	1.01 (–1.23 to 3.34)	–1.22 (–3.59 to 1.16)
**General health**						
Good health	–1.29 (–2.12 to –0.68)	–1.35 (–2.39 to –0.51)	0.05 (–1.13 to 1.24)	–0.16 (–0.35 to 0.04)	0.01 (–0.17 to 0.17)	–0.17 (–0.43 to 0.09)
Poor health	–1.03 (–1.76 to –0.54)	0.59 (–1.66 to 2.81)	–1.62 (–3.94 to 0.70)	–0.18 (–0.68 to 0.32)	0.13 (–0.04 to 0.27)	–0.31 (–0.84 to 0.21)

© 2026 Al Hasan SM et al. *JAMA Network Open*.

**Physical activity**						
Yes	–1.20 (–2.20 to –0.47)	–1.24 (–2.93 to 0.09)	0.03 (–1.71 to 1.78)	–0.11 (–0.35 to 0.13)	0.09 (–0.11 to 0.28)	–0.20 (–0.51 to 0.11)
No	–1.24 (–1.93 to –0.75)	–0.24 (–1.13 to 0.57)	–1.01 (–2.04 to 0.03)	–0.30 (–0.64 to –0.04)	–0.13 (–0.37 to 0.07)	–0.16 (–0.53 to 0.21)
**Smoking status**						
Current smoker	–1.53 (–2.23 to –1.02)	–1.05 (–1.92 to –0.32)	–0.48 (–1.48 to 0.52)	–0.13 (–0.89 to 0.47)	0.14 (–0.74 to 0.93)	–0.27 (–1.35 to 0.81)
Former smoker	–1.24 (–1.79 to –0.79)	–1.39 (–2.55 to –0.46)	0.15 (–1.01 to 1.31)	–0.36 (–0.69 to –0.04)	–0.11 (–0.29 to 0.05)	–0.25 (–0.61 to 0.12)
Never smoked	–1.30 (–3.07 to 0.50)	–1.15 (–2.19 to –0.25)	–0.15 (–2.19 to 1.88)	–0.15 (–0.50 to 0.14)	–0.07 (–0.53 to 0.34)	–0.08 (–0.62 to 0.47)
**Alcohol intake**						
Yes	–1.16 (–1.85 to –0.65)	–1.15 (–2.12 to –0.36)	–0.02 (–1.08 to 1.05)	–0.14 (–0.34 to 0.01)	–0.01 (–0.26 to 0.23)	–0.14 (–0.44 to 0.17)
No	–1.32 (–2.52 to –0.40)	–0.99 (–2.21 to 0.08)	–0.33 (–1.89 to 1.23)	–0.14 (–0.45 to 0.09)	0.01 (–0.11 to 0.11)	–0.15 (–0.45 to 0.14)

[a]The average biennial percentage change (ABPC) was calculated as a geometric weighted average of the biennial percent changes of various segments of mammography use prevalence trends in the US from 2012–2022 and 2012–2018.

[b]To assess the potential impact of the COVID-19 pandemic on mammography use prevalence trends, we calculated and compared the ABPCs for two periods: 2012–2022 (including the pandemic years) and 2012–2018 (pre-pandemic); the differences between "including the pandemic years" and "pre-pandemic period".

[c]Other races included Native Hawaiian/other Pacific Islander and races and ethnicities not specified and termed as "Others" in the BRFSS dataset.

[d]Employed for wages includes the employed for wages and the self-employed.

[e]Out of work group included women who were out for work, both less than 1 year and more than 1 year.

[f]Other employment groups include students, the retired, and women unable to work.

[g]Separated includes divorced, widowed, and separated women.

[h]Not married includes never married and a member of an unmarried couple.

AN stands for Alaska Native; HCP stands for healthcare provider.

© 2026 Al Hasan SM et al. *JAMA Network Open*.

eTable 9: Differences in biennial changes in mammography use by state between 2012–2022 and 2012–2018

	40–49 years, ABPC (95% CI)[a]			50–74 years, ABPC (95% CI)[a]		
**States**	**2012–2022**	**2012–2018**	**Differences[b]**	**2012–2022**	**2012–2018**	**Differences[b]**
Alabama	–0.23 (–1.15 to 0.52)	–0.26 (–2.16 to 1.43)	0.04 (–1.94 to 2.02)	–0.10 (–0.97 to 0.62)	0.19 (–1.46 to 1.69)	–0.29 (–2.06 to 1.48)
Alaska	–2.07 (–3.69 to –0.62)	–5.82 (–11.72 to –1.79)	3.75 (–1.45 to 8.95)	–0.62 (–1.19 to –0.03)	–1.79 (–3.10 to –0.79)	1.18 (–0.11 to 2.47)
Arizona	–0.50 (–2.07 to 0.98)	0.62 (–2.77 to 3.77)	–1.13 (–4.74 to 2.48)	–0.17 (–0.68 to 0.27)	–0.30 (–2.46 to 1.70)	0.13 (–2.00 to 2.26)
Arkansas	0.13 (–1.17 to 1.40)	–1.36 (–3.36 to 0.48)	1.49 (–0.82 to 3.80)	0.90 (0.51 to 1.32)	0.99 (–0.20 to 2.25)	–0.09 (–1.38 to 1.21)
California	–3.09 (–5.78 to –1.27)	–2.74 (–5.13 to –0.55)	–0.34 (–3.56 to 2.87)	–0.83 (–1.13 to –0.66)	–0.29 (–0.86 to 0.23)	–0.54 (–1.13 to 0.06)
Colorado	–1.05 (–3.52 to 0.96)	–1.44 (–4.75 to 1.41)	0.39 (–3.42 to 4.19)	–0.34 (–0.87 to 0.08)	–0.41 (–1.83 to 0.98)	0.07 (–1.41 to 1.55)
Connecticut	–0.63 (–0.94 to –0.36)	–0.75 (–1.08 to –0.44)	0.13 (–0.31 to 0.56)	–0.35 (–1.02 to 0.11)	–0.03 (–0.93 to 0.93)	–0.32 (–1.41 to 0.77)
Delaware	–1.69 (–2.97 to –0.70)	–1.17 (–3.29 to 0.56)	–0.52 (–2.75 to 1.71)	–0.65 (–1.59 to 0.08)	–0.20 (–1.66 to 1.08)	–0.45 (–2.05 to 1.16)
District of Columbia	–1.79 (–3.28 to –0.39)	–2.14 (–4.78 to 0.54)	0.35 (–2.67 to 3.37)	–0.75 (–1.72 to 0.11)	–0.85 (–2.52 to 0.89)	0.10 (–1.83 to 2.03)
Florida	–0.50 (–1.49 to 0.51)	1.76 (–0.43 to 4.28)	–2.26 (–4.81 to 0.30)	0.25 (–0.08 to 0.51)	1.11 (0.35 to 1.97)	–0.85 (–1.72 to 0.01)
Georgia	–1.28 (–3.11 to 0.32)	–1.43 (–3.56 to 0.63)	0.15 (–2.56 to 2.86)	–0.55 (–1.00 to –0.14)	–0.34 (–1.25 to 0.65)	–0.21 (–1.26 to 0.83)
Hawaii	–1.21 (–1.56 to –0.83)	–0.15 (–0.77 to 0.48)	–1.06 (–1.78 to –0.33)	–0.22 (–0.80 to 0.41)	1.14 (0.56 to 1.87)	–1.36 (–2.25 to –0.47)
Idaho	–0.30 (–2.77 to 2.50)	–2.19 (–4.93 to 0.18)	1.89 (–1.78 to 5.56)	0.14 (–0.73 to 1.00)	–0.72 (–2.18 to 0.58)	0.86 (–0.77 to 2.48)
Illinois	–1.94 (–3.05 to –1.04)	0.15 (–0.22 to 0.54)	–2.08 (–3.16 to –1.01)	–0.12 (–0.91 to 0.52)	0.18 (0.06 to 0.29)	–0.30 (–1.02 to 0.43)
Indiana	–0.65 (–2.40 to 1.06)	–0.53 (–4.83 to 3.39)	–0.12 (–4.58 to 4.34)	0.84 (0.11 to 1.58)	1.08 (0.31 to 1.86)	–0.24 (–1.31 to 0.83)
Iowa	–1.28 (–2.66 to 0.10)	–0.97 (–1.87 to –0.12)	–0.31 (–1.95 to 1.32)	0.08 (–0.42 to 0.57)	0.04 (–0.68 to 0.79)	0.04 (–0.84 to 0.92)
Kansas	–1.25 (–1.70 to –0.82)	–2.41 (–5.61 to 0.28)	1.16 (–1.82 to 4.14)	–0.66 (–0.96 to –0.39)	–0.97 (–1.52 to –0.49)	0.32 (–0.27 to 0.91)
Kentucky	–0.45 (–4.57 to 2.51)	–1.26 (–9.91 to 6.35)	0.81 (–8.06 to 9.67)	–0.26 (–1.71 to 0.74)	0.24 (–1.19 to 1.49)	–0.51 (–2.32 to 1.31)
Louisiana	0.00 (–1.84 to 1.54)	–1.70 (–3.33 to –0.55)	1.70 (–0.49 to 3.89)	0.58 (0.25 to 0.87)	0.77 (0.08 to 1.37)	–0.19 (–0.90 to 0.53)
Maine	–1.75 (–2.55 to –1.19)	–2.25 (–4.26 to –0.98)	0.50 (–1.27 to 2.28)	–0.20 (–0.42 to 0.03)	–0.61 (–0.95 to –0.33)	0.42 (0.04 to 0.80)
Maryland	–1.10 (–2.14 to –0.11)	–0.49 (–1.56 to 0.70)	–0.61 (–2.13 to 0.91)	–0.11 (–0.50 to 0.23)	–0.61 (–0.99 to –0.21)	0.50 (–0.04 to 1.04)
Massachusetts	–2.00 (–2.45 to –1.75)	–2.06 (–2.55 to –1.80)	0.06 (–0.45 to 0.58)	–0.48 (–0.77 to –0.31)	–0.67 (–1.37 to –0.28)	0.20 (–0.40 to 0.79)
Michigan	–0.29 (–0.56 to –0.09)	–1.33 (–1.81 to –0.92)	1.04 (0.54 to 1.55)	–0.42 (–0.85 to –0.03)	–0.36 (–1.39 to 0.62)	–0.06 (–1.14 to 1.02)
Minnesota	–1.53 (–2.50 to –0.64)	–1.50 (–2.25 to –0.74)	–0.03 (–1.23 to 1.16)	–0.46 (–1.10 to 0.12)	–0.18 (–0.61 to 0.27)	–0.28 (–1.04 to 0.47)
Mississippi	1.27 (0.53 to 2.04)	0.72 (–1.41 to 2.96)	0.55 (–1.76 to 2.86)	–0.04 (–1.25 to 1.08)	–0.67 (–1.59 to 0.09)	0.64 (–0.80 to 2.07)
Missouri	0.30 (–0.71 to 1.37)	0.11 (–0.87 to 1.00)	0.18 (–1.22 to 1.59)	–0.10 (–0.82 to 0.61)	–0.41 (–1.91 to 0.90)	0.32 (–1.26 to 1.89)
Montana	–0.79 (–2.19 to 0.31)	–2.98 (–6.84 to –0.41)	2.19 (–1.26 to 5.64)	0.48 (0.12 to 0.78)	0.85 (–0.08 to 1.64)	–0.37 (–1.29 to 0.55)
Nebraska	–0.70 (–2.02 to 0.32)	–0.09 (–1.14 to 0.76)	–0.61 (–2.11 to 0.90)	0.18 (–0.25 to 0.53)	–0.07 (–1.34 to 0.96)	0.26 (–0.96 to 1.47)
Nevada	–0.20 (–5.39 to 5.27)	1.95 (–4.43 to 8.21)	–2.16 (–10.42 to 6.11)	–0.02 (–1.27 to 1.24)	–0.20 (–0.71 to 0.28)	0.18 (–1.17 to 1.53)
New Hampshire	–1.93 (–4.63 to –0.16)	–2.05 (–4.35 to –0.11)	0.12 (–2.96 to 3.21)	–0.60 (–1.12 to –0.15)	–0.48 (–1.45 to 0.39)	–0.12 (–1.16 to 0.92)
New Jersey	–1.53 (–2.67 to –0.90)	–3.12 (–4.44 to –2.34)	1.59 (0.22 to 2.96)	–0.30 (–0.62 to –0.04)	0.35 (–0.55 to 0.99)	–0.65 (–1.47 to 0.17)
New Mexico	–2.66 (–4.51 to –1.44)	–2.77 (–7.39 to 0.48)	0.11 (–4.11 to 4.32)	–0.09 (–0.66 to 0.37)	–0.24 (–0.43 to –0.07)	0.15 (–0.40 to 0.69)
New York	–1.32 (–1.90 to –0.56)	–2.00 (–3.43 to –0.37)	0.68 (–0.99 to 2.35)	0.15 (–0.96 to 1.49)	0.48 (–0.98 to 2.46)	–0.33 (–2.44 to 1.78)
North Carolina	–0.32 (–0.85 to –0.01)	0.62 (–2.43 to 2.94)	–0.94 (–3.65 to 1.78)	–0.10 (–0.49 to 0.19)	–0.18 (–0.83 to 0.28)	0.08 (–0.57 to 0.73)
North Dakota	–0.02 (–3.32 to 2.83)	–1.49 (–7.30 to 3.72)	1.47 (–4.84 to 7.78)	0.29 (–0.22 to 0.83)	–0.02 (–2.67 to 2.76)	0.31 (–2.45 to 3.07)

© 2026 Al Hasan SM et al. *JAMA Network Open*.

Ohio	–1.60 (–2.15 to –1.10)	0.92 (0.60 to 1.23)	–2.51 (–3.12 to –1.90)	–0.11 (–0.73 to 0.54)	–0.08 (–0.71 to 0.50)	–0.02 (–0.90 to 0.86)
Oklahoma	–0.58 (–2.17 to 0.71)	0.13 (–3.22 to 2.93)	–0.72 (–4.11 to 2.68)	–0.07 (–0.63 to 0.40)	1.18 (–0.44 to 2.69)	–1.25 (–2.9 to 0.40)
Oregon	–0.85 (–3.05 to 1.59)	–0.54 (–5.27 to 4.73)	–0.32 (–5.83 to 5.19)	0.45 (–0.13 to 1.03)	0.41 (–0.77 to 1.59)	0.03 (–1.28 to 1.35)
Pennsylvania	0.13 (–0.38 to 0.47)	–0.42 (–1.34 to 0.20)	0.55 (–0.33 to 1.43)	–0.20 (–1.91 to 0.89)	–0.63 (–3.11 to 0.95)	0.42 (–2.04 to 2.89)
Rhode Island	–1.19 (–2.68 to –0.11)	–1.27 (–4.49 to 1.22)	0.08 (–3.05 to 3.20)	0.06 (–0.14 to 0.26)	0.26 (0.01 to 0.52)	–0.20 (–0.53 to 0.13)
South Carolina	–0.22 (–1.18 to 0.42)	–2.06 (–3.62 to –0.74)	1.84 (0.19 to 3.48)	0.48 (0.19 to 0.70)	0.17 (–0.29 to 0.62)	0.30 (–0.22 to 0.82)
South Dakota	1.93 (0.72 to 2.98)	2.06 (0.69 to 3.33)	–0.14 (–1.87 to 1.60)	–0.04 (–0.83 to 0.58)	0.37 (–0.32 to 1.07)	–0.41 (–1.40 to 0.57)
Tennessee	–0.74 (–1.23 to –0.30)	–2.04 (–6.64 to 1.37)	1.30 (–2.73 to 5.34)	–0.24 (–0.94 to 0.32)	–0.16 (–0.99 to 0.56)	–0.08 (–1.08 to 0.91)
Texas	–1.22 (–4.06 to 0.84)	–0.55 (–3.00 to 1.36)	–0.67 (–3.95 to 2.61)	0.10 (–0.94 to 0.95)	0.23 (–2.06 to 2.02)	–0.12 (–2.37 to 2.12)
Utah	–1.33 (–2.00 to –0.69)	–3.41 (–8.55 to 0.73)	2.08 (–2.61 to 6.76)	–0.04 (–1.15 to 0.87)	0.26 (–1.69 to 2.07)	–0.30 (–2.44 to 1.84)
Vermont	–2.99 (–3.70 to –2.34)	–4.13 (–5.05 to –3.48)	1.15 (0.11 to 2.18)	–0.82 (–1.55 to –0.19)	–0.94 (–1.41 to –0.53)	0.12 (–0.69 to 0.93)
Virginia	–0.89 (–2.53 to 0.66)	–1.06 (–2.87 to 0.63)	0.17 (–2.20 to 2.53)	–0.38 (–0.62 to –0.14)	0.04 (–0.53 to 0.63)	–0.42 (–1.05 to 0.21)
Washington	–2.05 (–2.93 to –1.27)	–3.67 (–6.24 to –1.63)	1.62 (–0.84 to 4.07)	–0.21 (–0.49 to 0.07)	–0.30 (–1.03 to 0.38)	0.09 (–0.67 to 0.84)
West Virginia	0.30 (–2.18 to 2.69)	1.06 (–2.47 to 4.61)	–0.75 (–5.05 to 3.54)	–0.07 (–0.75 to 0.56)	–0.38 (–2.10 to 1.34)	0.31 (–1.53 to 2.14)
Wisconsin	–1.30 (–1.77 to –0.83)	0.08 (–2.30 to 2.30)	–1.39 (–3.74 to 0.96)	–0.11 (–0.38 to 0.18)	–1.06 (–1.69 to –0.50)	0.95 (0.29 to 1.60)
Wyoming	0.42 (–1.44 to 2.25)	–0.79 (–4.50 to 2.87)	1.20 (–2.92 to 5.32)	–0.34 (–1.89 to 0.98)	–0.02 (–2.94 to 2.79)	–0.32 (–3.52 to 2.88)

[a]The average biennial percentage change (ABPC) was calculated as a geometric weighted average of the biennial percent changes of various segments of mammography use prevalence trends in the US from 2012–2022 and 2012–2018.

[b]To assess the potential impact of the COVID-19 pandemic on mammographic screening prevalence trends, we calculated and compared the ABPCs for two periods: 2012–2022 (including the pandemic years) and 2012–2018 (pre-pandemic); the differences between “including the pandemic years” and “pre-pandemic period”.

© 2026 Al Hasan SM et al. *JAMA Network Open*.

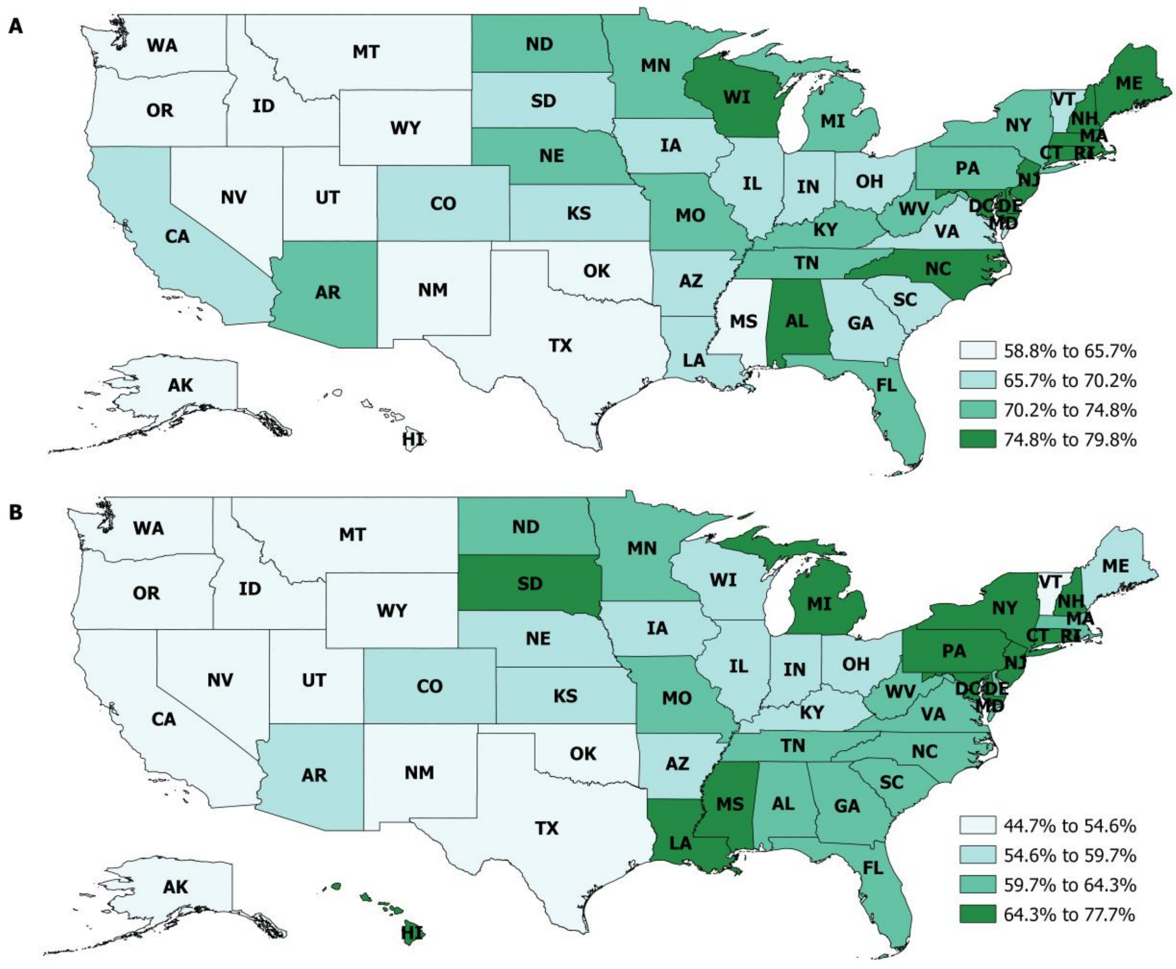

eFigure 1: Mammography use prevalence among women aged 40–49 years in 2002 and 2022

Prevalence was categorized into quartiles. (A) In 2002, the quartiles of mammography use prevalence for women aged 40–49 years were 58.8%–65.7% (1st quartile), 65.7%–70.2% (2nd quartile), 70.2%–74.8% (3rd quartile), and 74.8%–79.8% (4th quartile). (B) In 2022, the tertiles for women aged 40–49 years were 44.7%–54.6% (1st quartile), 54.6%–59.7% (2nd quartile), 59.7%–64.3% (3rd quartile), and 64.3%–77.7% (4th quartile).

© 2026 Al Hasan SM et al. *JAMA Network Open*.

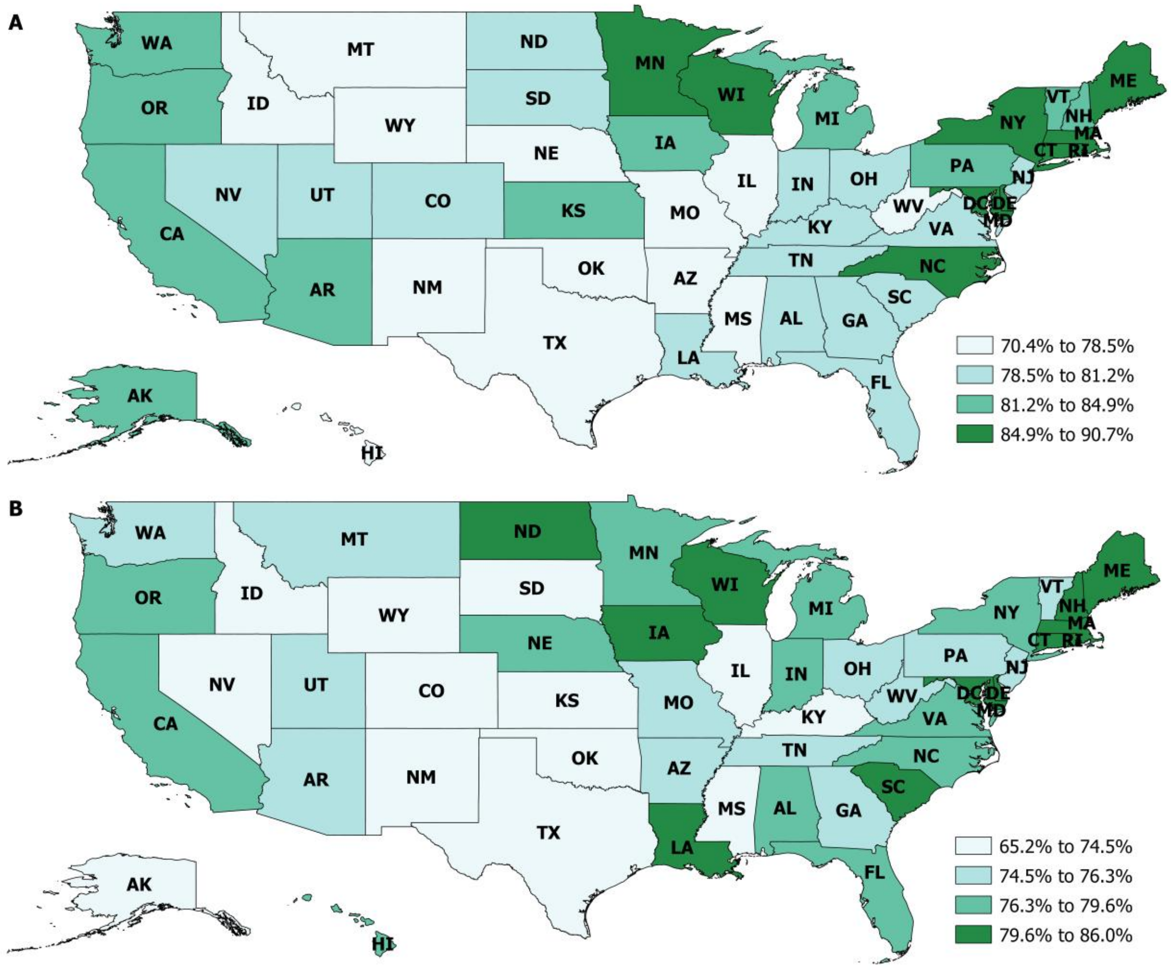

eFigure 2: Mammography use prevalence among women aged 50–74 years in 2002 and 2022

Prevalence was categorized into quartiles. (A) In 2002, the quartiles of mammography use prevalence for women aged 50–74 years were 70.4%–78.5% (1st quartile), 78.5%–81.2% (2nd quartile), 81.2%–84.9% (3rd quartile), and 84.9%–90.7% (4th quartile). (B) In 2022, the tertiles for women aged 50–74 years were 65.2%–74.5% (1st quartile), 74.5%–76.3% (2nd quartile), 76.3%–79.6% (3rd quartile), and 79.6%–86.0% (4th quartile).

© 2026 Al Hasan SM et al. *JAMA Network Open*.

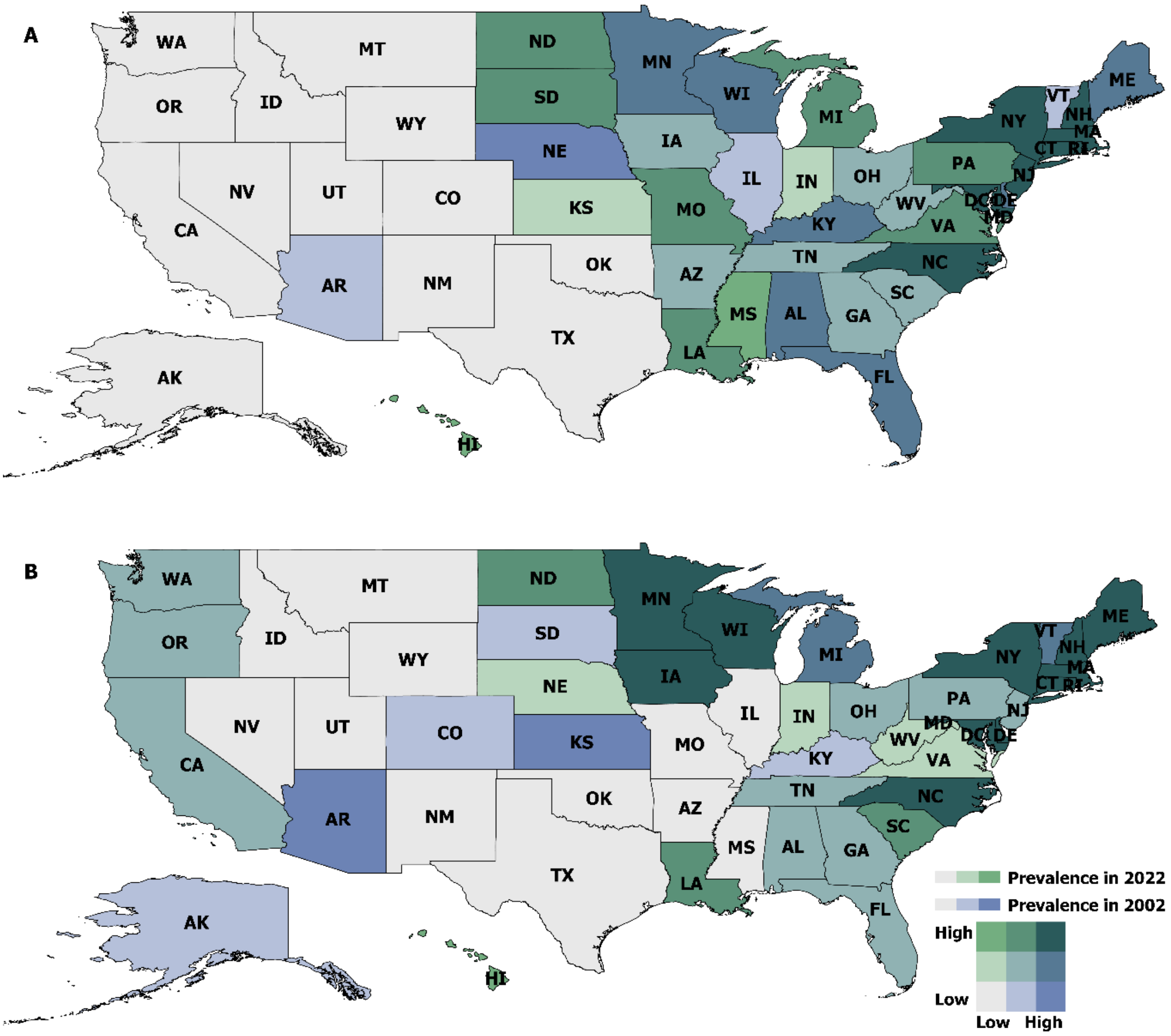

eFigure 3: Bivariate spatial patterns of mammography use in 2002 and 2022 among women aged 40–49 and 50–74 years

The prevalence was categorized into tertiles. (A) For women aged 40–49 years, the mammography use prevalence tertiles in 2002 were 58.8%–67.1% (low), 67.1%–72.2% (medium), and 72.2%–79.8% (high); and in 2022 were 44.7%–55.5% (low), 55.5%–62.0% (medium), and 62.0%–77.7% (high). (B) For women aged 50–74 years, the mammography use prevalence tertiles in 2002 were 70.4%–79.6% (low), 79.6%–82.2% (medium), and 82.2%–90.7% (high); and in 2022 were 65.2%–75.0% (low), 75.0%–78.1% (medium), and 78.1%–86.0% (high).

© 2026 Al Hasan SM et al. *JAMA Network Open*.

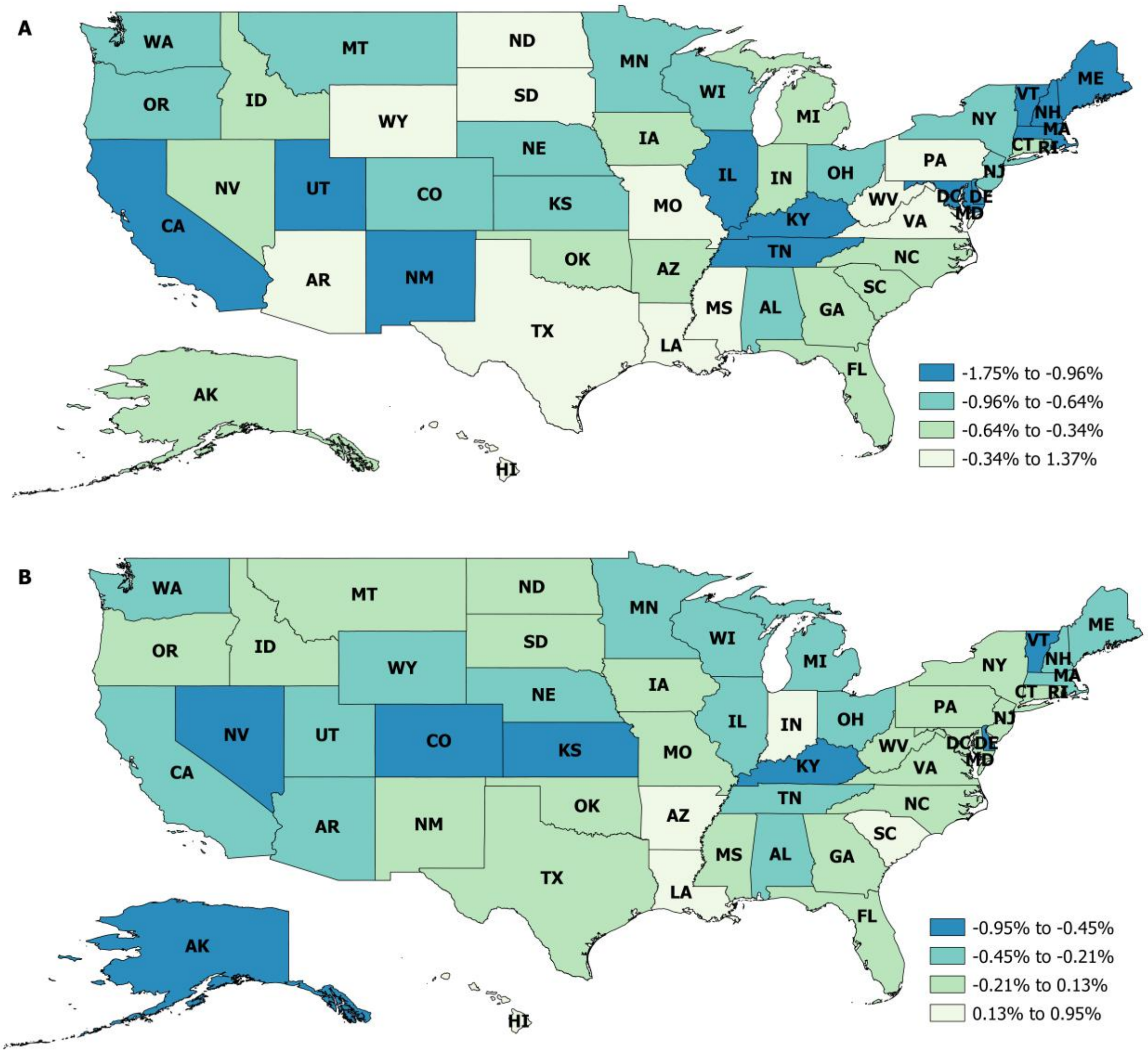

eFigure 4: Average biennial percent changes in mammography use among women aged 40–49 and 50–74 years, 2002–2022

The biennial percent changes in mammography use prevalence were categorized as a quartile distribution of percentage changes per biennium. (A) For women aged 40–49 years during the entire period (2002–2022), states in the 1st quartile had an average biennial reduction rate from 1.75% to 0.96%, states in the 2nd quartile had an average biennial reduction rate of 0.96% to 0.64%, states in the 3rd quartile had an average biennial reduction rate of 0.64% to 0.34%, and states in the 4th quartile had an average biennial change from –0.34% to 1.37%. (B) For women aged 50–74 years during the entire period (2002–2022), states in the 1st quartile had an average biennial reduction rate from 0.95% to 0.45%, states in the 2nd quartile had an average biennial reduction rate of 0.45% to 0.21%, states in the 3rd quartile had an average biennial change from –0.21% to 0.13%, and states in the 4th quartile had an average biennial increasing rate from 0.13% to 0.95%.

© 2026 Al Hasan SM et al. *JAMA Network Open*.
