# Supplementary material for: Trends in Mammography Use Among Women Aged 40 to 74 Years in the US, 2002-2022
Source: JAMA Netw Open. 2026 Mar 26;9(3):e263529. doi: 10.1001/jamanetworkopen.2026.3529 (PMC13022741; doi:10.1001/jamanetworkopen.2026.3529)
Supplement: Supplement 2. — Data Sharing Statement [file jamanetwopen-e263529-s002.pdf]

Data Sharing Statement

Al Hasan. Trends in Mammography Use Among Women Aged 40 to 74 Years in the US, 2002-2022. *JAMA Netw Open*. Published March 26, 2026. doi:10.1001/jamanetworkopen.2026.3529

Data
Data available: Yes
Data types: Data (not involving human participants)
How to access data: The data used in this study are publicly available from the BRFSS, administered by the Centers for Disease Control and Prevention. These data and codebooks can be accessed at https://www.cdc.gov/brfss
When available: With publication

Supporting Documents
Document types: None

Additional Information
Who can access the data: The data is publicly available at any time.
Types of analyses: For any purpose
Mechanisms of data availability: Without investigator support.
